# Molecular and cellular insight into *Escherichia coli* SslE and its role during biofilm maturation

**DOI:** 10.1038/s41522-022-00272-5

**Published:** 2022-02-25

**Authors:** Paula M. Corsini, Sunjun Wang, Saima Rehman, Katherine Fenn, Amin Sagar, Slobodan Sirovica, Leanne Cleaver, Charlotte J. C. Edwards-Gayle, Giulia Mastroianni, Ben Dorgan, Lee M. Sewell, Steven Lynham, Dinu Iuga, W. Trent Franks, James Jarvis, Guy H. Carpenter, Michael. A. Curtis, Pau Bernadó, Vidya C. Darbari, James A. Garnett

**Affiliations:** 1 Centre for Host-Microbiome Interactions, Faculty of Dental, Oral & Craniofacial Sciences, King’s College London, London, UK; 2 School of Biological and Behavioural Sciences, Queen Mary University of London, London, UK; 3 Centre de Biologie Structurale, Université de Montpellier, INSERM, CNRS, Montpellier, France; 4 Centre for Oral Bioengineering, Barts and The London School of Medicine and Dentistry, Queen Mary University of London, London, UK; 5 Diamond Light Source Ltd., Diamond House, Harwell Science and Innovation Campus, Didcot, Oxfordshire UK; 6 Proteomics Facility, Centre of Excellence for Mass Spectrometry, King’s College London, London, UK; 7 Department of Physics, University of Warwick, Coventry, UK; 8 Randall Division of Cell and Molecular Biophysics and Centre for Biomolecular Spectroscopy, King’s College London, London, UK

**Keywords:** Biofilms, Bacteriology, Pathogens

## Abstract

*Escherichia coli* is a Gram-negative bacterium that colonises the human intestine and virulent strains can cause severe diarrhoeal and extraintestinal diseases. The protein SslE is secreted by a range of pathogenic and commensal *E. coli* strains. It can degrade mucins in the intestine, promotes biofilm maturation and it is a major determinant of infection in virulent strains, although how it carries out these functions is not well understood. Here, we examine SslE from the commensal *E. coli* Waksman and BL21 (DE3) strains and the enterotoxigenic H10407 and enteropathogenic E2348/69 strains. We reveal that SslE has a unique and dynamic structure in solution and in response to acidification within mature biofilms it can form a unique aggregate with amyloid-like properties. Furthermore, we show that both SslE monomers and aggregates bind DNA in vitro and co-localise with extracellular DNA (eDNA) in mature biofilms, and SslE aggregates may also associate with cellulose under certain conditions. Our results suggest that interactions between SslE and eDNA are important for biofilm maturation in many *E. coli* strains and SslE may also be a factor that drives biofilm formation in other SslE-secreting bacteria.

## Introduction

*Escherichia coli* is a primary coloniser of the lower intestinal tract of humans and other warm‐blooded animals. While many strains are considered beneficial to the host and help to maintain a healthy immune system, virulent strains are the cause of severe diarrhoeal diseases, including haemorrhagic colitis, and extraintestinal diseases, such as neonatal meningitis, urinary tract infections, sepsis and pneumonia^1^. A wide range of pathogenic *E. coli* strains, and some commensals, use a *Vibrio*-like type II secretion system (T2SS) to translocate the protein SslE across their outer membrane and onto their extracellular surface^2–7^. These include the Waksman (W), enterotoxigenic (ETEC), enterohemorrhagic (EHEC), enteropathogenic (EPEC), enteroaggregative (EAEC), enteroinvasive (EIEC) and neonatal meningitis *E. coli* (NMEC) strains. SslE is required for full virulence in a rabbit model of EPEC infection^4^ and as a surface-exposed antigen, SslE has shown great promise as a broadly protective vaccine candidate against a wide range of *E. coli* pathotypes^3,6,8,9^.

SslE interacts with mucosal membranes in the host intestine where it can degrade mucins^6,10–12^, a family of heavily *O*-linked glycosylated proteins and the primary constituents of mucus^13^. This provides nutrients during bacterial growth but also facilitates the penetration of pathogenic *E. coli* strains through the gut mucosa to access host cells for efficient colonisation and targeting of toxins/effectors. Furthermore, SslE is important for mediating the maturation of EPEC biofilms^4^; microbial aggregations encased within a self-produced extracellular matrix comprised of exopolysaccharides, adhesive proteins and nucleic acids^14^. When *E. coli* is released into the environment through faeces or wastewater effluent it can survive for long periods within complex biofilm communities^15,16^, and these are fundamental for both the environmental ecology of *E. coli* but also for successful colonisation of the intestinal tract^17^. However, the specific molecular mechanisms that SslE uses to promote ecology and/or disease are not well understood.

**Fig. 1:**
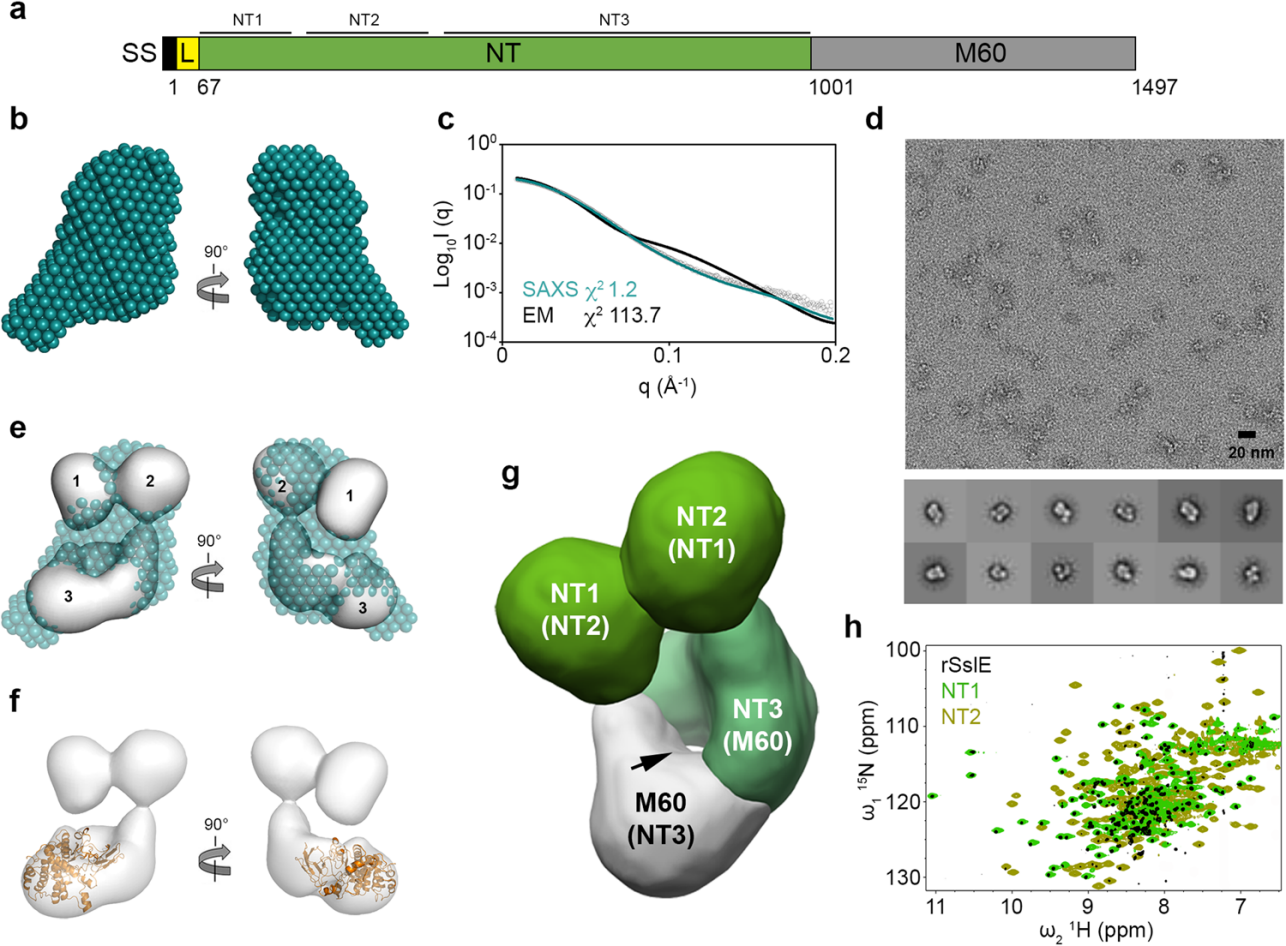
Structural features of monomeric SslE. **a** Schematic of SslE from *E. coli* W with mature sequence numbers and structural features annotated. SS: periplasmic signal sequence; L: flexible linker; NT: unique N-terminal region; M60: peptidase/mucinase domain. **b** SAXS bead model of rSslE at pH 7.4. **c** Fit of the SAXS bead model (teal line) and negative-stain TEM map (black line) of rSslE to the rSslE SAXS data (black open circles) with χ^2^ of 1.2 and 113.7, respectively. **d** Representative negative-stain TEM micrograph of rSslE (pH 7.4) at ×50,000 nominal magnification with representative 2D classifications. Scale bar represents 20 nm. **e** Overlay of the 22 Å resolution TEM map (grey) and SAXS bead model (teal) with the three defined regions in SslE highlighted. **f** Docking of an SslE M60 domain homology model (orange) into the TEM map. **g** TEM map of rSslE coloured based on domain organisation. Possible alternative assignments of the NT1 and NT2 domains in regions 1 and 2, and the NT3 and M60 domains in region 3 are shown in parentheses. The NT3-M60 interdomain channel is highlighted with an arrow. **h** TROSY ^1^H^15^N-HSQC spectrum of rSslE (black) overlaid with ^1^H^15^N-HSQC spectra of the SslE NT1 (light green) and NT2 (olive green) domains.

SslE is a ~165 kDa lipoprotein composed of an N-terminal periplasmic localisation sequence and lipobox motif, an unstructured ~5 kDa region, a ~110 kDa region with no significant primary sequence homology to any other known protein, and a ~50 kDa M60-like aminopeptidase domain at its C-terminus^18^ (Fig. 1a). M60-like domains are metalloproteases that contain a zinc-binding HExxH motif and an additional conserved catalytic glutamate residue, which cleaves the peptide backbone of mucin-like substrates. These and other related enzymes have been identified in both prokaryotic and eukaryotic microbes that interact with host mucosal membranes^18^ and structures of proteoglycan complexes suggest that interactions with both the mucin peptide and *O*-linked carbohydrate side chains are important for specificity^19,20^. The peptidase activity of the SslE M60 domain is effective for the degradation of major mucins of the intestine (e.g. MUC2, MUC3, MUC5AC)^6,10–12^, however, very little is known as to how SslE interacts with these mucins or the function of the remaining SslE sequence.

In this study, we provide new insight into the global structure of SslE and propose a molecular mechanism by which SslE can promote biofilm maturation. Using transmission electron microscopy (TEM), small-angle X-ray scattering (SAXS), nuclear magnetic resonance (NMR) spectroscopy and biochemical analyses we show that SslE is formed of three defined regions (NT1, NT2 and NT3-M60), and is dynamic in solution. We also demonstrate that SslE undergoes conformational changes under acidic conditions, and this leads to the formation of higher-order structures with amyloid-like properties. We directly observe acidification within mature *E. coli* W biofilms and determine that both monomeric and aggregated forms of SslE can bind extracellular DNA (eDNA) in *E. coli* W biofilms, and under some conditions SslE aggregates may also associate with extracellular cellulose. We propose that SslE acts like a hub and provides a cross-linking function in biofilms, and this is likely a general mechanism for SslE in all SslE-secreting bacteria.

## Results

### Overall structural features of SslE

To understand the role of SslE in *E. coli* biofilms, we first initiated a structural analysis of recombinant SslE from *E. coli* W, minus the N-terminal lipidation motif and adjacent disordered region (rSslE; residues 67 to 1497; UniProt ID E0IW31) (Fig. 1a). This was produced in *E. coli* BL21 and purified by nickel-affinity and size-exclusion chromatography. Small-angle X-ray scattering (SAXS) coupled with size-exclusion chromatography (SEC-SAXS) was then used to get shape information for rSslE at pH 7.4. Guinier analysis provided a radius of gyration (*R*_g_) of 4.03 ± 0.05 nm and examination of the distance distribution function (*P(r)*) suggested a maximum particle dimension (*D*_max_) of 14.1 nm (Supplementary Fig. 1 and Supplementary Table 1). Calculation of the particle molecular mass (151 kDa) was consistent with a monomer in solution (theoretical mass 160 kDa). Ab initio dummy residue reconstruction generated 20 reproducible models with an average normalised spatial discrepancy (NSD) score between reconstructions of 0.56 and a χ^2^ fit between calculated and experimental solution scattering of 1.2 (Fig. 1b, c). The averaged low-resolution bead model suggested that rSslE has a slightly elongated structure, however, examination of the Kratky plot indicated that rSslE is a dynamic particle (Supplementary Fig. 1) and no obvious domain features could be assigned.

Negative-stain TEM data were next collected for rSslE at pH 7.4 and we performed single-particle analysis to generate an initial low-resolution structure, with overall dimensions of ~8 × 8 × 11.5 nm (Fig. 1d and Supplementary Fig. 2). Here, three well-defined regions could be identified: two adjacent globular domains (regions 1 and 2) with approximate diameters of 4 nm and 4.5 nm, respectively, and a torus-shaped structure (region 3) with a ~15 Å wide central channel and approximate overall dimensions of 4.5 × 6.5 × 9 nm (Fig. 1e). The superposition of the SAXS bead model with the TEM envelope showed some disagreement, with an apparent reduction of bead volume in all three regions of the SAXS model. Although this observation suggests the presence of internal dynamics in rSslE, in line with its Kratky plot, the low-resolution nature of SAXS data does not allow discarding a different origin. Rigid body docking of a homology model of the M60 domain, based on the IMPa M60 metalloprotease from *Pseudomonas aeruginosa* (PDB ID code 5kdv)^19^, into the TEM envelope indicated that the C-terminus of SslE is located within region 3, most likely at the base (Fig. 1f), and would not fit the density within regions 1 and 2. This suggested that the remaining density in region 3 is composed of the SslE sequence directly N-terminal to the M60 domain and these two regions pack against one another to form a stable structure around an interdomain channel. Furthermore, from this model the M60 HExxH active site motif faces into the channel, and this could be an important feature for its mucolytic activity.

Secondary structure analysis^21^ of the SslE sequence indicated two potential interdomain boundaries within the N-terminal region and we predicted that these represented either region 1 or 2, located at the extreme N-terminus (NT) of SslE. We therefore renamed these as the NT1 (residues 67–211) and NT2 (residues 230–425) domains, respectively, and renamed the remaining sequence as NT3 (residues 426–1000) (Fig. 1g). The NT1 and NT2 domains, along with the NT1-NT2 region (residues 67–425) and NT3-M60 core (region 3; residues 426–1497) were then produced in *E. coli* BL21 and purified by nickel-affinity and size-exclusion chromatography. Examination of these constructs using analytical gel filtration, nuclear magnetic resonance (NMR) spectroscopy and SEC-SAXS showed that they were well folded (Supplementary Figs. 3 and 4), and supported NT1, NT2 and NT3-M60 being defined structural boundaries within SslE.

We then examined intact rSslE by NMR, but only ~250 strong resonances were observed in a ^1^H^15^N transverse relaxation optimised spectroscopy (TROSY) HSQC spectrum, with the remaining ~1200 peaks being either very weak or completely absent (Fig. 1h). However, NMR relaxation measurements of these intense peaks provided an estimated correlation time consistent with an ~80 kDa domain (τ_c_ ∼ 35 ns at 37 °C). Comparison of rSslE with ^1^H^15^N-HSQC spectra from SslE sub-domains also clearly showed that the rSslE NMR spectrum is dominated by the NT1 domain but with minor contributions from the NT2 domain (Fig. 1h and Supplementary Fig. 5). This implied that the resonances from the NT1 and NT2 domains originated from intact rSslE and further supported that this region has a significant independent motion with respect to the larger NT3-M60 core.

### SslE forms aggregates with amyloid-like properties

**Fig. 2:**
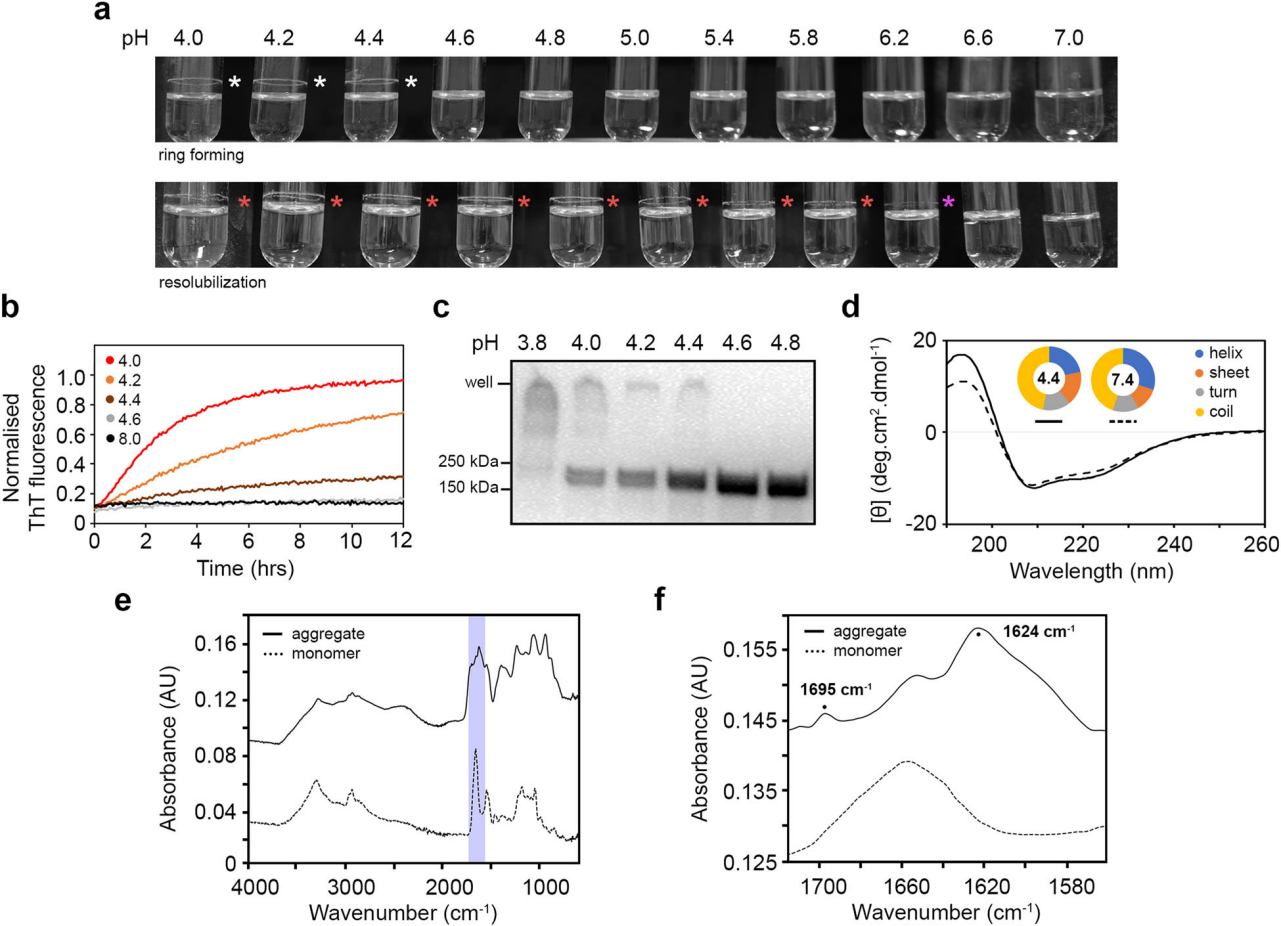
Characterisation of SslE aggregates. **a** Purified rSslE forms a clear ring of aggregated protein when incubated in the citrate-phosphate buffer between pH 4.0 and 4.4 (upper panel; white asterisks). The ring formed above the liquid line is due to shaking of the sample and it resists solubilisation up to pH 6.2 once formed (lower panel; purple asterisk), although some loss of protein band is observed after pH 5.8 (lower panel; red asterisks). **b** Increase in ThT fluorescence emission on binding to rSslE aggregates formed in citrate-phosphate buffer over pH 4.0–8.0. **c** Immunoblot of rSslE incubated in citrate-phosphate buffer from pH 3.8 to 4.8 and detected with anti-His_6_ antibody. rSslE present in the well represents an aggregated protein. Blots were derived from the same experiment and processed in parallel. **d** Far-UV CD spectra of soluble rSslE at pH 4.4 (solid line) and 7.4 (dashed line). Secondary structure analysis is shown in the doughnut chart. **e** ATR FT-MIR spectrum of monomeric rSslE and aggregates with the amide I region shaded in violet. **f** Expanded ATR FT-MIR amide I region displaying the relatively high and low-intensity absorption bands in the aggregates but not monomeric spectrum at ~1624 and 1695 cm^−1^, respectively, characteristic of an anti-parallel β-sheet structure. Spectra are offset in the absorbance axis for clarity.

Functional amyloids are important proteinaceous structures in biofilms^22^ and analysis of the SslE primary sequence had previously highlighted several regions across the protein that may form amyloid-like structures^23^. Furthermore, environmental pH plays a major role in initiating the formation of amyloid-like structures in *Staphylococcus aureus*, *Enterococcus faecalis* and *Streptococcus mutans* biofilms^24–26^, and we speculated that pH may also stimulate similar structural changes in SslE. We therefore incubated rSslE across the pH range 4.0–7.0 and observed a ring of protein deposited on the walls of the tube at pH ≤ 4.4, which resisted solubilisation up to pH 6.2 (Fig. 2a). Examination of the ring by SDS-PAGE revealed a major high molecular weight species that was retained in the well (Supplementary Fig. 6). Subsequent analysis of this by mass spectrometry identified peptides that spanned the complete sequence of rSslE and indicated that the aggregate contained intact protein, rather than a degradation product (Supplementary Fig. 6). We next used the amyloid diagnostic dye Thioflavin-T (ThT) to assess the formation of rSslE aggregates in solution over time. We observed large increases in ThT fluorescence emission again across the pH range 4.0–4.4, but no change was detected when rSslE was incubated above pH 4.4 (Fig. 2b). Likewise, when rSslE was boiled and treated with sodium dodecyl sulphate (SDS) and then analysed by anti-His_6_ immunoblotting, stable high molecular weight species were observed over the same pH range (Fig. 2c). However, we also observed two distinct bands of monomeric SslE, which suggested that it had been proteolytically cleaved at its N-terminus. Together these data indicated that SslE may form amyloid-like structures at low pH and so this was further scrutinised with additional biophysical analysis.

A main feature of amyloids is the presence of a cross-β-sheet structure^27^ and so we first analysed the secondary structure of soluble rSslE using far-UV circular dichroism (CD) spectroscopy. This suggested that at pH 7.4 rSslE is composed of ~30% α-helix, 12% β-sheet and 45% coil, while under acidic conditions and prior to aggregation, there is an ~8% loss of helical secondary structure with a ~5% increase in β-sheet structure (Fig. 2d and Supplementary Table 2). We next used attenuated total reflectance (ATR) Fourier transform mid-infrared (FT-MIR) spectroscopy to probe the secondary structure composition of soluble rSslE at pH 7.4 and rSslE fibres at pH 4.4. Examination of the amide I absorption band showed peaks at ~1656 cm^−1^ and 1624 cm^−1^, respectively (Fig. 2e). The shift in the amide I absorption band to a lower frequency indicated that a larger and more rigid structure had assembled under acid conditions. Furthermore, the presence of both a relatively high-intensity absorption band at ~1624 cm^−1^ and a lower intensity band at ~1695 cm^−1^ in the pH 4.4 sample was indicative of an increase in anti-parallel β-sheet structure in rSslE aggregates^28^ and is consistent with previous FT-MIR based characterisations of anti-parallel β-sheet amyloid fibres^29^ (Fig. 2f).

### SslE aggregates display a fibrillar morphology

**Fig. 3:**
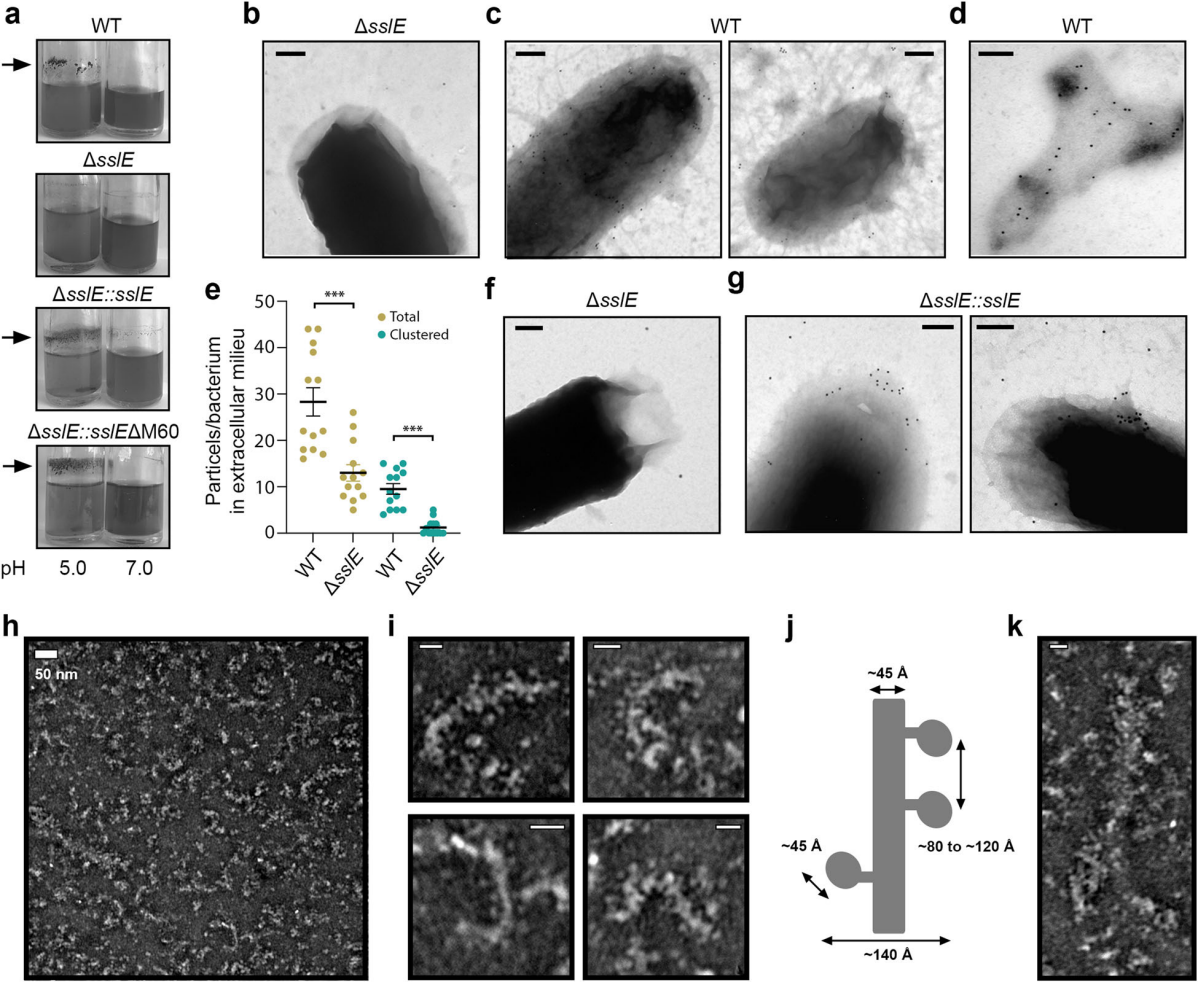
TEM analysis of *E. coli* W SslE aggregations. **a** Accumulation of SslE aggregates on the side of glass tubes containing overnight cultures grown in LB media at pH 5.0 or 7.0. Arrows indicate Congo red-stained ring containing SslE. **b**–**g** SslE localisation upon secretion from *E. coli* W strains assessed by immunoelectron microscopy. Bacteria were reacted with primary α-rSslE antibodies and secondary gold-labelled antibodies on carbon-coated nickel grids and then negatively stained with uranyl acetate. **b**, **f** *E. coli* W Δ*sslE* mutant washed in citrate-phosphate buffer at pH 5.0 or 4.0, respectively. **c** Wild-type W strain washed in citrate-phosphate buffer at pH 5.0 with α-rSslE antibodies reacting with the bacterial surface and secreted fibrous material. **d** Image highlighting clustering of antibodies to an extracellular aggregate. **e** Analysis of total and clustered (≤25 nm between particles) α-rSslE antibodies reacting with aggregates in the extracellular milieu of wild-type and Δ*sslE* mutant bacteria at pH 5.0. ^***^P < 0.001; verses Δ*sslE* mutant by two-tailed Student’s test and error bars are the standard error of the mean. **g** Δ*sslE::sslE* W strain washed in citrate-phosphate buffer at pH 4.0, again with α-rSslE antibodies reacting with the bacterial surface and secreted fibrous material. Scale bar is equivalent to 200 nm. **h** TEM analysis of negatively stained rSslE aggregates. Two major species are observed. **i** Smaller filaments have an average width of ∼4.5 nm and are coated with globular structures of ~4.5 nm in diameter. Scale bar, 20 nm. **j** Schematic describing the overall dimensions of the smaller SslE fibre species. **k** Larger species of SslE aggregates appear as ~20–40 nm wide structures with variable lengths. Scale bar, 20 nm.

Congo red is a dye that can bind to amyloids and extracellular polymeric substances (EPS) in biofilms, including curli and cellulose. When we grew *E. coli* W strain overnight in liquid culture at pH 5.0, we observed a clear ring on the wall of the tube that retained Congo red, but this ring was less apparent when grown at pH 7.0 (Fig. 3a). Examination of this ring by SDS-PAGE again revealed a large molecular weight species retained in the wells and mass spectrometry analysis identified intact SslE as the predominant protein (Supplementary Fig. 6). We then created an isogenic Δ*sslE* knockout mutant in *E. coli* W, but this strain was not able to form a substantial ring at either pH (Fig. 3a). However, complementation with a plasmid containing intact *sslE* (Δ*sslE::sslE*) or SslE with a truncated C-terminus (Δ*sslE::sslEΔM60*; residues 1–1000) restored both SslE secretion and ring formation at pH 5.0 (Fig. 3a and Supplementary Fig. 7).

Examination of wild-type *E. coli* W at pH 5.0 by immunoelectron microscopy revealed gold-labelled polyclonal anti-rSslE antibodies associated with the bacterial outer membrane, which was not seen in the Δ*sslE* mutant (Fig. 3b, c). The fibrous material was also observed in the wild-type strain in the extracellular milieu and emanating away from the bacterial surface (Fig. 3c, d). Although antibody binding to these aggregates was less dramatic, the number of these single and clustered gold particles (measured up to 1.0 µm from each bacterium) was significantly higher in the wild-type strain compared with the Δ*sslE* mutant (Fig. 3b–e and Supplementary Fig. 8). Inspection of the *E. coli* W Δ*sslE::sslE* complemented strain under the same conditions produced less clustering of anti-rSslE antibodies, however, a wild-type phenotype was observed when cells were incubated at pH 4.0 prior to fixation and staining (Fig. 3f, g). As a control, wild-type bacteria were also incubated with anti-CsgA (curli fibre) antibodies and there was no accumulation of gold particles on or away from the bacterial surface (Supplementary Fig. 9). Although these data indicated that SslE may form fibres upon secretion from *E. coli* under acidic conditions, we were not able to differentiate between an accumulation of antibodies with aggregates and surface-associated or secreted monomeric SslE.

Negative-stain TEM was then used to visualise purified rSslE fibres and this revealed two common morphologies (Fig. 3h). The first form appeared as short single, flexible fibres with a core structure measuring ~4.5 nm wide by ~110 nm in length and decorated with globular structures ~4.5 nm in diameter (Fig. 3i, j). The second morphology again resembled a fibrous material but measured between ~20 and 40 nm in width by 200–300 nm in length (Fig. 3k) and appeared to be an aggregation of the smaller fibres. Further analysis of rSslE aggregates in solution by real-time multi-angle light scattering (RT-MALS) supported these observations (Supplementary Fig. 10). We measured a smaller species with an average molecular mass of 5.4 × 10^3^ kDa (±0.4 × 10^3^) and an average *R*_g_ of 137.5 nm (±1.7), and a larger more polydisperse species with an average molecular mass of 12 × 10^3^ kDa (±0.8 × 10^3^) and an average *R*_g_ of 139.8 nm (±1.5). Examination of rSslE fibres using solid-state NMR (ssNMR) showed individual narrow signals which confirmed that this was not an artefactual aggregation (Supplementary Fig. 11). We assigned ~40 ordered residues to specific residue types and this pattern suggested that these ordered regions were localised to more than one site across the SslE sequence.

### Kinetics of SslE aggregation

**Fig. 4:**
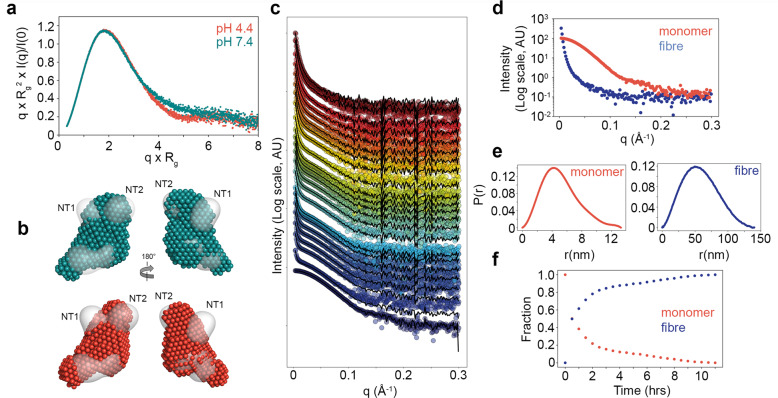
Kinetics of SslE aggregation. **a** SAXS normalised Kratky plots for rSslE at pH 4.4 and 7.4. **b** Overlay of rSslE SAXS bead models at pH 4.4 (red) and 7.4 (teal) with rSslE TEM map (grey). NT1 and NT2 domains are highlighted. **c** COSMiCS decomposition of the time-dependent rSslE SAXS fibrillation data, assuming that the whole dataset can be described with two co-existing components (monomer and fibre, panel **d**) with different relative populations (**f**). SEC-SAXS curve for rSslE at pH 4.4 (bottom curve) is also shown for *t* = 0 and used to define the curve of the monomeric component. Experimental curves are displayed in colour and arbitrarily displaced along the *y* axis for a better inspection. Black curves are the result of the COSMiCS optimisation. The excellent agreement with the experimental data testifies the accuracy of COSMiCS decomposition. **e** Shape distribution (*P(r)*) functions derived from COSMiCS decomposed scattering curves for rSslE monomer (red) and aggregates (blue). **f** Relative populations of the two species of rSslE derived from the COSMiCS analysis over time.

We took advantage of SEC-SAXS to probe the global shape of rSslE at pH 4.4 prior to fibre formation and both Guinier analysis (*R*_*g*_ 3.92 ± 0.05 nm) and examination of the distance distribution function (*P(r)*) (*D*_*max*_ 13.7 nm) produced very similar values to rSslE at pH 7.4 (Supplementary Fig. 12 and Supplementary Table 1). However, the presence of better-defined features in the SAXS profile at acidic pH and the differences at larger q-values in the normalised Kratky plots were indicative of rSslE becoming more rigid under acidic conditions (Fig. 4a and Supplementary Fig. 13). Ab initio dummy residue reconstruction produced 20 models with an average NSD score of 0.45 and a χ^2^ fit between calculated and experimental solution scattering of 1.0 (Fig. 4b and Supplementary Fig. 14). Comparison of the averaged bead models indicated an increase in bead volume within the NT1 and NT2 domains and adjacent NT3 region at pH 4.4, while little change was observed in the NT3-M60 core, which supported an increase in rigidity under acidic conditions that could trigger aggregation.

We then exploited the additive nature of SAXS along with its ability to examine structures over a large size range to study the kinetics of fibre formation by rSslE. We acquired SAXS data at pH 4.4 during the course of aggregation for 11 h and the resulting 23 profiles displayed a systematic upwards intensity increase, indicating the presence of large aggregates in solution (Fig. 4c). The appearance of these large species occurred relatively quickly as the upwards intensity increase was already observed after 1 h and the increase of aggregated species was concomitant to the loss of the SAXS features linked to the monomeric species. Principal Component Analysis (PCA) indicated that the dataset could be described as a two-component system (Supplementary Fig. 15) and we decomposed the time-dependent dataset along with the SAXS profile of the monomer, obtained using SEC-SAXS, with COSMiCS. COSMiCS uses a chemometric approach to decompose SAXS data sets in a model-free manner to derive the pure SAXS curves of the co-existing species and their relative populations, which we used here to report on the fibrillation kinetics^30,31^. The COSMiCS decomposition, assuming the co-existence of two components, was able to adequately fit all the SAXS profiles with an average <χ^2^ > of 1.5, although some deviations from the perfect fit were observed for the SAXS curves measured in the first 3 h (Fig. 4c). This observation suggested the presence of a small population of a third species in the initial steps of fibrillation but this putative third species could not be captured when considering a three-species decomposition with COSMiCS.

The extracted profile of the smallest species, and the subsequently derived *R*_g_ (3.92 nm) and *D*_*max*_ (13.3 nm) values, were almost identical to the SEC-SAXS profile for rSslE at pH 4.4 (Fig. 4d, e and Supplementary Table 1), and this indicated that SslE is in a monomeric form at the beginning of the fibrillation process. The extracted SAXS profile of the second component displayed the typical features of a large particle and *P(r)* analysis indicated it had an *R*_*g*_ of ~51.1 nm and *D*_*max*_ of ~140 nm (Fig. 4d, e and Supplementary Table 1). Furthermore, fractal fit analysis^32,33^ of the decomposed SAXS profiles also indicated that rSslE aggregates into a structure that is fractal in nature (Supplementary Fig. 16). The radius of the primary particle was determined to be ~3.5 nm while the mass fractal and surface fractal dimensions were determined to be 2.9 and 3.0, respectively.

Examination of the relative populations of the two species derived from the COSMiCS decomposition over time showed that after 30 min from initiating the measurements, the aggregated species already represented 50% of the molar fraction (Fig. 4f). From this time point, this population continued to grow and reached a plateau after ~10 h. As the SAXS fibrillation data at the 10 h time point had shown depletion of monomeric rSslE but also had reduced fractal properties compared with later time points (Fig. 4f), we discarded the low q region and used this data to carry out analysis of the fibre asymmetric unit. Geometrical shape analysis suggested that rSslE fibres form a cylinder with a radius 3.2 nm and height 16.5 nm, and cross-sectional analysis indicated a repetitive unit with a cross-sectional *R*_*g*_ (*R*_*c*_) of 4.78 nm. The cross-sectional *P*(*r*)_*C*_ gave a cross-sectional *D*_*max*_ of 14.0 nm. Together these data supported our observations of single and clustered rSslE fibres derived from negative-stain TEM and MALS.

### Biofilm maturation supports polymerisation of SslE

**Fig. 5:**
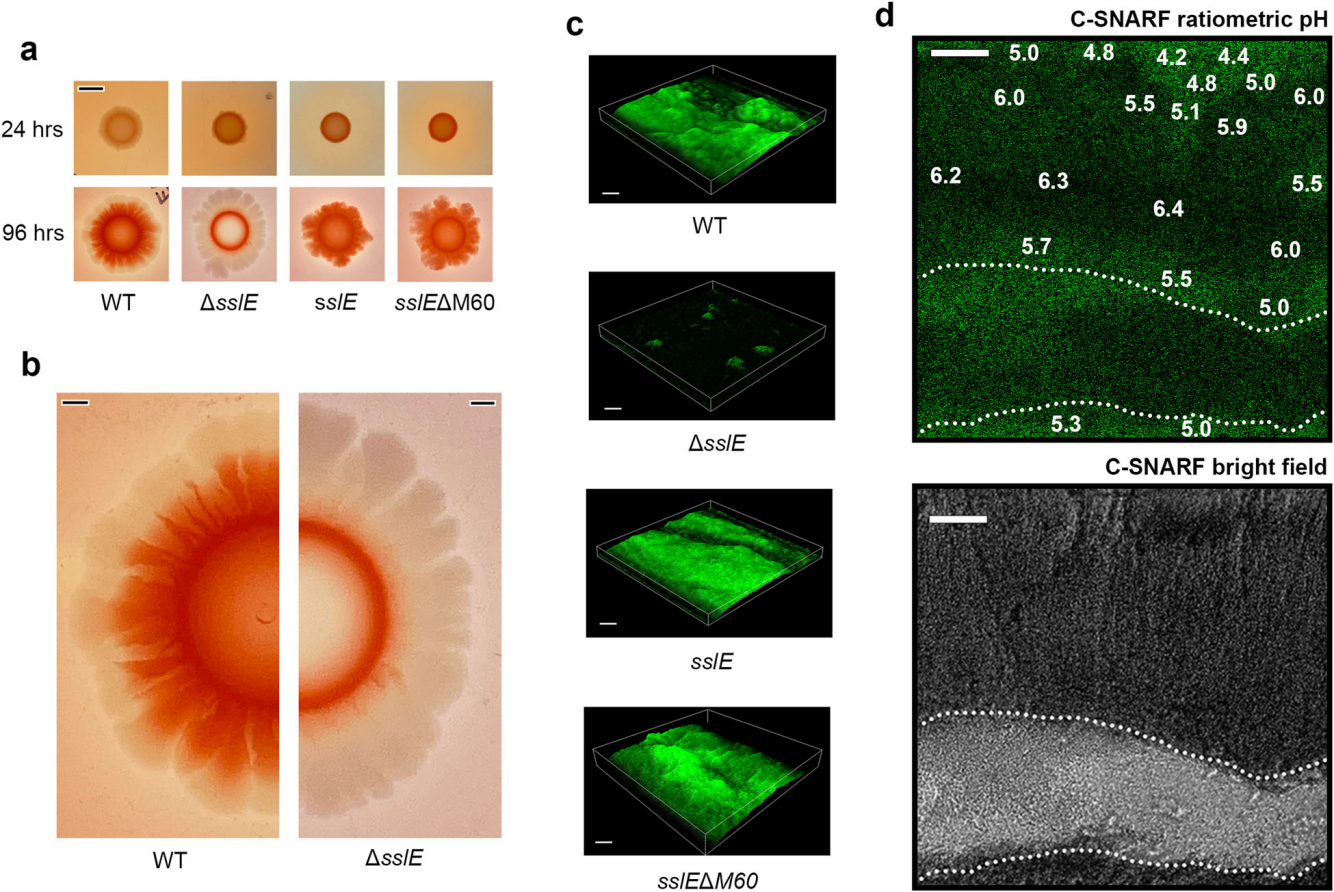
Analysis of SslE-dependent biofilm formation by *E. coli* W strain. **a** Macrocolony biofilm phenotype of wild-type *E. coli* W and derivatives on LB Congo red agar medium after 24 and 96 h of growth. The derivatives *sslE* and *sslE*ΔM60 represent *trans*-complementation of intact *sslE* (Δ*sslE::sslE*) or *sslE* minus its M60 domain (Δ*sslE::sslE*ΔM60), respectively, into the *E. coli* W Δ*sslE* mutant. Scale bar = 5 mm. **b** Zoomed-in image of the wild-type *E. coli* W and Δ*sslE* mutant. Scale bar = 2 mm. **c** CLSM images of wild-type *E. coli* W biofilms and *E. coli* W Δ*sslE* mutant and *trans*-complementation with *sslE* and *sslE*ΔM60 stained with FM 1-43 (green). Scale bar represents 20 μm. **d** The pH across wild-type *E. coli* W biofilms grown for 96 h was monitored ratiometrically using C-SNARF-4 (green). pH values were calculated over ~30 μm^2^ boxes and pH values for representative regions within the biofilm fringes and centres are annotated. Dotted line outlines water channel within the biofilm, where pH values are not shown. Scale bar represents 20 μm. All data are representative of at least three independent experiments.

As SslE aggregates had been shown to bind Congo red, we used this dye to assess the role of SslE during the formation of *E. coli* W strain macrocolony biofilms. When we compared the Δ*sslE* mutant cultured on LB Congo red agar at 37 °C for 24 h to its parental wild-type strain we observed no major differences (Fig. 5a). After 96 h both strains displayed a lobated morphology but in the mutant the dye was retained as a compact ring within the centre of the colony, while in the wild-type strain the dye radiated out from the middle of the colony towards the edge (Fig. 5a, b). However, complementation of the Δ*sslE* mutant with intact *sslE* or *sslEΔM60* was able to recover macrocolony morphology similar to that of the parental wild-type (Fig. 5a).

We reasoned that if SslE aggregates are formed in macrocolonies and can bind Congo red dye, SslE must experience extracellular pH values <5.0 during the development of these biofilms. To test this, we first grew *E. coli* W biofilms using a microfluidic system under continuous flow and assessed their overall morphology using confocal laser scanning microscopy (CLSM). While biofilms grown for 48 h produced a relatively homogenous lawn of bacterial growth, after 96 h there were clear signs of maturation, with a significant increase in biofilm mass, structural heterogeneity, and the presence of water channels (Fig. 5c and Supplementary Fig. 17). We then examined the *E. coli* W Δ*sslE* mutant under these conditions, but in line with previous reports in EPEC strain E2348/69^4^, it was unable to develop structures beyond microcolonies (Fig. 5c). Furthermore, complementation of the mutant with either intact *sslE* or *sslEΔM60* was again able to restore wild-type biofilm morphology. Together with our previous observations, this indicated that the C-terminal M60 domain is not required for translocation of SslE through its T2SS and it is dispensable for biofilm development, at least under these conditions.

We then examined pH distribution across *E. coli* W biofilms using CLSM coupled with the cell-impermeant fluorescent ratiometric probe seminaphthorhodafluor-4F 5-(and-6) carboxylic acid (C-SNARF-4). After growing *E. coli* W for 24 h under continuous flow, we observed pH values between 6.0 and 6.3 across biofilms (Supplementary Fig. 18), however, after 96 h, lower pH values were also recorded (Fig. 5d). Although across most biofilms examined, we detected pH values between approximately 5.0 and 6.0, clearly defined microenvironments were also observed with pH values that ranged from 4.2 and 4.8.

### Analysis of EPS in *E. coli* macrocolony biofilms

We speculated that during the maturation of biofilms, SslE would interact with other components of the biofilm matrix and may contribute to the structural integrity of these communities. Cellulose, curli, polymeric β-1,6-linked N-acetylglucosamine (PNAG) and colanic acid are major EPS in *E. coli* biofilms^34^ and we examined which of these are produced in macrocolonies from several *E. coli* strains under conditions that support the production of SslE. *E. coli* W, ETEC H10407 and EPEC E2348/69 strains were studied as they had previously been shown to secrete SslE^4,5,11^, while *E. coli* BL21 (DE3) strain was expected to secrete SslE based on genomic analysis^5^. In addition, we also incorporated several other control strains. The *E. coli* K12 strain BW25113 was used as it does not produce SslE or cellulose due to deletion of several *T2SS* genes and mutations in its cellulose biosynthesis genes^5,35^, respectively, and we created an isogenic Δ*sslE* knockout mutant in ETEC H10407. We also examined the published EPEC E2348/69 Δ*sslE*, E2348/69 Δ*csgA* (curli defective) and E2348/69 Δ*csgA*/Δ*bcsA* (curli and cellulose defective) knockout mutant strains^4,36^, and the BW25113 Δ*wcaF* (colanic acid defective) and BW25113 Δ*pgaC* (PNAG defective) knockout mutant strains from the Keio collection^37^.

**Fig. 6:**
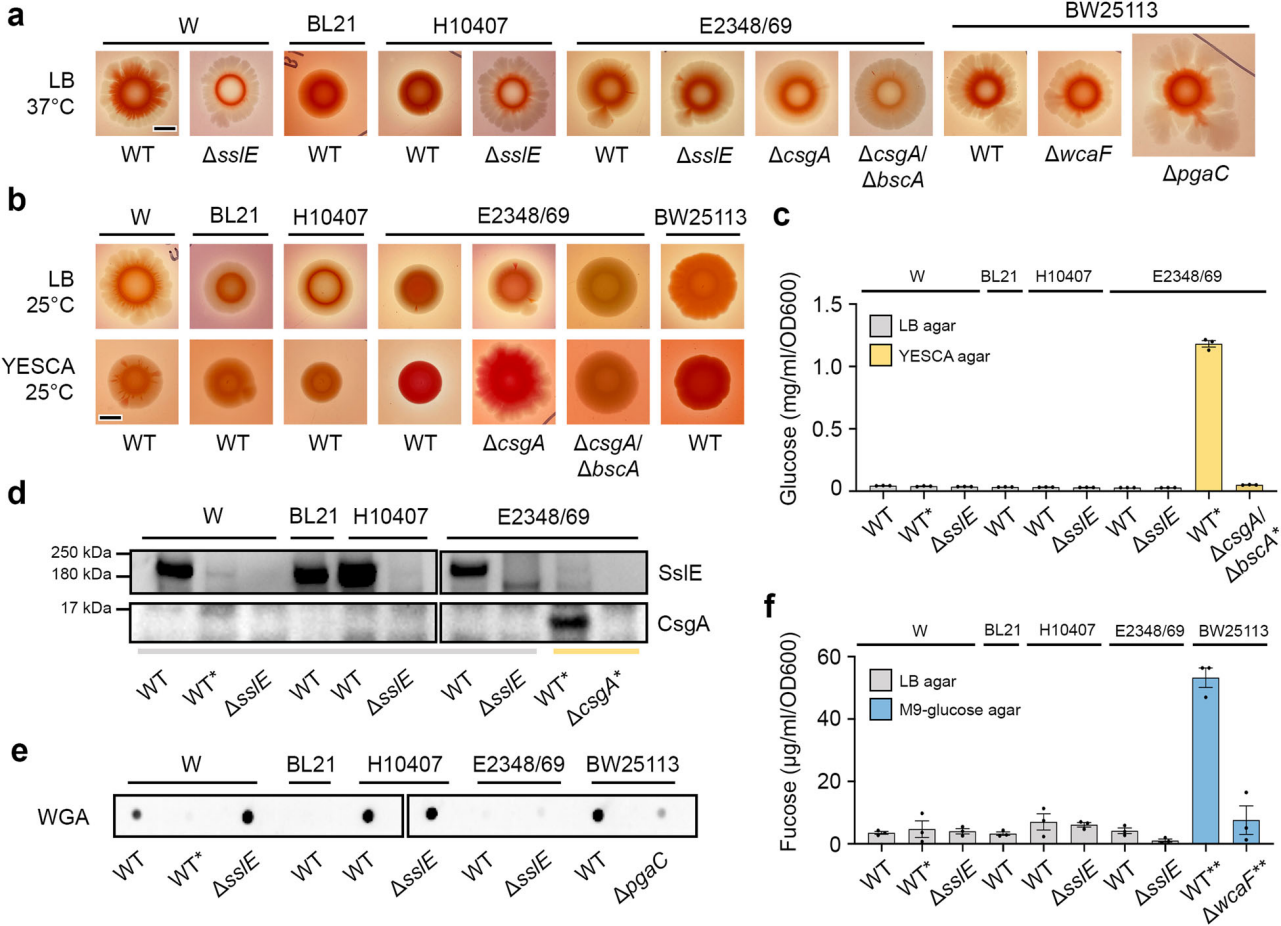
EPS production in *E. coli* macrocolony biofilms. Analysis and quantification of EPS from macrocolony biofilms of wild-type *E. coli* W, BL21 (DE3), H10407 E2348/69 and BW25113 strains and their derivatives after 96 h growth. **a** Macrocolony phenotypes of strains grown at 37 °C on LB Congo red agar medium, **b** 25 °C on LB Congo red agar medium and 25 °C on YESCA Congo red agar medium. Scale bar represents 5 mm. **c** Cellulose production after digestion with cellulase from strains grown on LB agar (grey) or YESCA agar (yellow) at 37 °C (no asterisks) or 25 °C (*). **d** Immunoblot analysis of whole cells grown on LB agar (grey line) or YESCA agar (yellow line) at 37 °C (no asterisks) or 25 °C (*), detected with either anti-rSslE antibody or anti-CsgA antibody. **e** Dot-blot analysis of PNAG content from whole cells grown on LB agar at 37 °C (no asterisks) or 25 °C (*), detected with wheat germ agglutinin (WGA). **f** Quantification of colanic acid from strains grown on LB agar (grey line) or M9-glucose agar (blue line) at 37 °C (no asterisks), 25 °C (*) or 18 °C (**). Data are representative of at least three repeat experiments. All blots were derived from the same experiment and processed in parallel and error bars are the standard error of the mean.

We first cultured these strains on LB Congo red agar grown at 37 °C for 96 h (Fig. 6a). Both wild-type BL21 (DE3) and H10407 strains produced smooth and round colonies with an even distribution of dye, but deletion of the *sslE* gene in H10407 resulted in Congo red being retained as a ring in the centre of the colony. Colonies of wild-type E2348/69 strain formed circular and smooth colonies with slight lobing and with dye radiating out from the middle of the colony. However, little difference in colony morphology was observed between the E2348/69 Δ*sslE* and Δ*csgA* mutants and their parental wild-type strain, although the Δ*csgA*/Δ*bcsA* double mutant did show a reduction in Congo red intensity and localisation. Macrocolonies of wild-type BW25113 and its derivatives retained the dye as with the W and H10407 Δ*sslE* mutants, but while the wild-type and Δ*wcaF* mutant strains appeared similar in morphology, the Δ*pgaC* mutant showed significantly more lobation and grew approximately twice the size.

We next examined macrocolony morphologies after growth on LB Congo red agar and YESCA Congo red agar at 25 °C, conditions known to repress and induce the production of SslE and curli/cellulose, respectively (Fig. 6b). All wild-type strains on LB displayed an overall colony morphology similar to growth at 37 °C, however, W, BL21 (DE3) and H10407 strains displayed distribution of Congo red like their corresponding Δ*sslE* mutant. Wild-type BW25113 and E2348/69 strains on the other hand showed increased Congo red retention, but this was reduced in the E2348/69 Δ*csgA* and Δ*csgA*/Δ*bcsA* mutant strains. Examination of the W, BL21 (DE3), H10407 and E2348/69 Δ*csgA*/Δ*bcsA* strains on YESCA all showed a smooth and white morphology and implied that these strains do not produce curli or cellulose^38^. As anticipated, wild-type E2348/69 strain displayed a red, dry and rough morphology, indicative of both curli and cellulose production, while E2348/69 Δ*csgA* strain showed a pink, dry and rough morphology (cellulose only) and wild-type BW25113 appeared with a brown, dry and rough morphology (curli only)^38^. This indicated that under conditions that support the expression of *sslE*, neither curli nor cellulose are produced.

Extracellular levels of cellulose were next quantified by measuring glucose release after digestion with cellulase (Fig. 6c). After treatment all wild-type strains showed levels of glucose consistent with the E2348/69 Δ*csgA*/Δ*bcsA* double mutant strain. This was further supported through the examination of macrocolony growth in the presence of the cellulose stain calcofluor white, where only wild-type E2348/69 strain grown on YESCA agar at 25 °C displayed noticeable colony fluorescence with UV light (Supplementary Fig. 19). The relative expression of SslE and curli were then assessed by immunoblot analysis of macrocolonies (Fig. 6d). Production of SslE was observed in wild-type W, BL21 (DE3), H10407 and E2348/69 strains when grown at 37 °C on LB but not when grown at 25 °C on LB or YESCA agar. Conversely, the major curli subunit CsgA was only detected when E2348/69 strain was grown on YESCA agar at 25 °C. We then assessed the production of PNAG by probing partially purified extracellular carbohydrates using wheat germ agglutinin (Fig. 6e). Our results showed significant amounts of PNAG could be detected in W, H10407 and BW25113 strains when they were grown at 37 °C on LB agar, but no PNAG was detected in BL21 (DE3) or E2348/69 strains grown under the same conditions, or when the W strain grown at 25 °C on LB. We finally examined the presence of colanic acid in colonies by measuring fucose evolution after treatment with sulphuric acid (Fig. 6f). However, we could only detect colanic acid production when wild-type BW25113 strain was grown under nutrient limiting conditions at low temperature^39^ and not when strains were grown on LB agar at 37 °C.

### SslE associates with eDNA in biofilms

To examine whether rSslE can directly bind PNAG we first produced rSslE fibres at pH 4.0 and then isolated them in citrate-phosphate buffer at pH 6.0. At this pH, the structure of rSslE fibres would be retained, while monomeric rSslE would resist polymerisation (Fig. 2a). As a control, we used the C-terminal domain (CTD) of the *Porphyromonas gingivalis* gingipain protein RgpB, which was not expected to bind EPS and remained stable under these conditions (Supplementary Fig. 20). We then monitored the binding of rSslE fibres, rSslE monomers and RgpB-CTD to PNAG using a dot–blot overlay assay but could not detect any interaction (Supplementary Fig. 21). However, when we assayed the association of rSslE to immobilised cellulose discs produced by the bacterium *Komagataeibacter rhaeticus*, we detected a significant interaction with fibres, but not monomeric rSslE or RgpB-CTD (Supplementary Fig. 22). Nonetheless, the specificity of this binding was not investigated further as cellulose had not been detected in conditions that supported SslE production (Fig. 6a–c).

**Fig. 7:**
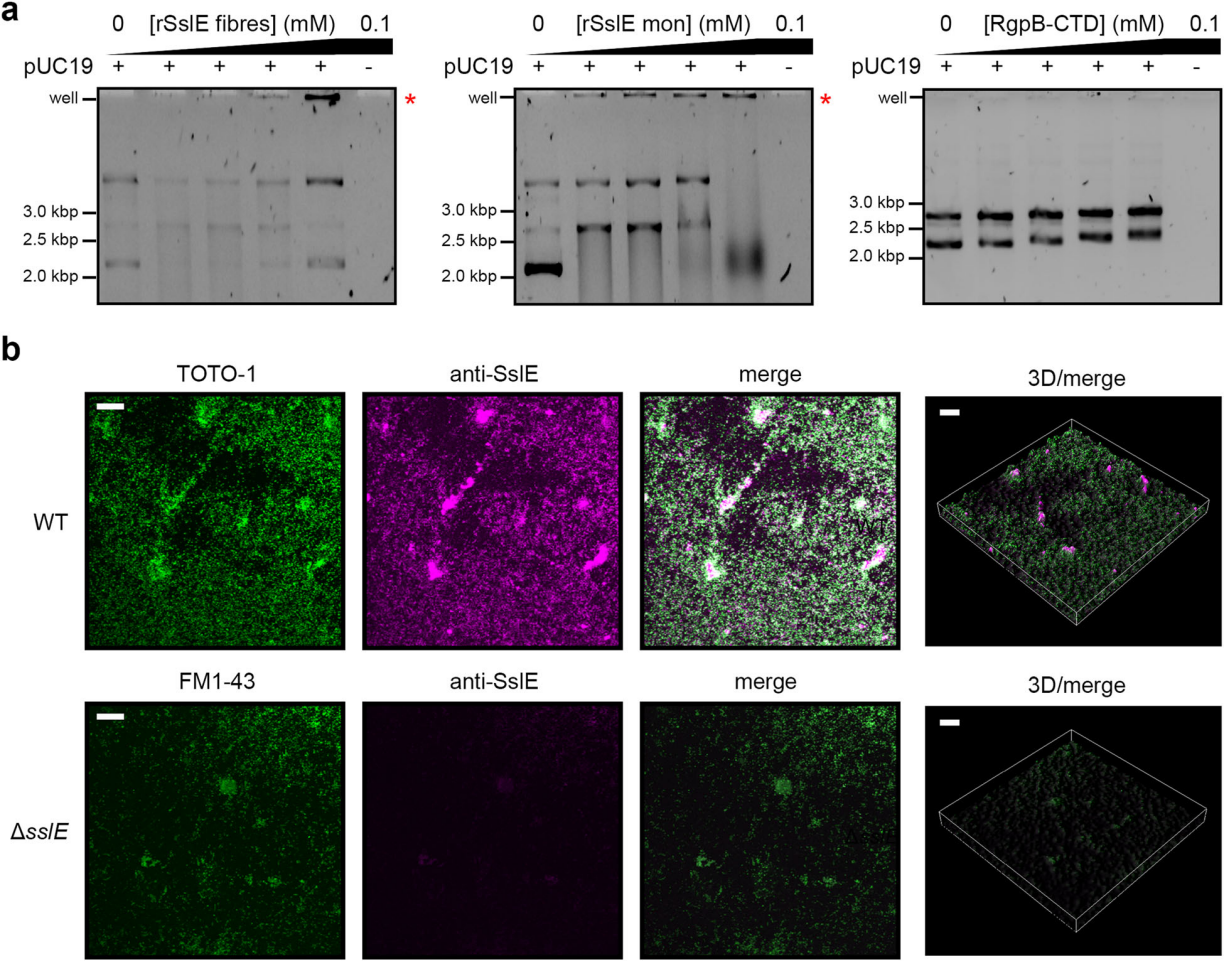
SslE associates with eDNA in *E. coli* W biofilms. **a** Electrophoretic mobility shift assay of rSslE monomers and fibres with pUC19 plasmid DNA substrate. *P. gingivalis* RgpB-CTD was used a negative control. Red asterisk (*) indicates the formation of a large protein–DNA complex retained in the wells. Gels were derived from the same experiment and processed in parallel. **b** Wild-type *E. coli* W biofilms were formed using a continuous flow cell system over 96 h. Immunofluorescence was performed where biofilms were incubated with α-rSslE antibodies followed by goat anti-rabbit conjugated Alexa Fluor 633 (magenta). Extracellular DNA and dead cells were stained with TOTO-1 (green). *E. coli* W Δ*sslE* strain was used as a control and again stained with α-rSslE/Alexa Fluor 633 (magenta) and the membrane dye FM 1–43 (green). Biofilms were visualised by CLSM. Scale bar is equivalent to 50 µm. All data are representative of at least three independent experiments.

Extracellular DNA (eDNA) is another key component of many biofilms and provides adhesion, structuring and chelation of cations^40^. We considered whether SslE could associate with eDNA and performed an electrophoretic mobility shift assay (EMSA) using the plasmid pUC19 as a DNA substrate. Plasmid DNA was incubated with increasing concentrations of either rSslE fibres, rSslE monomers or RgpB-CTD control at pH 6.0 and then analysed on agarose gels (Fig. 7a). Upon incubation of pUC19 with 100 μM rSslE fibres, a large protein–DNA complex was detected in the wells of the gel, but not at lower concentrations or when incubated with the control. Likewise, when pUC19 was incubated with 12.5 μM monomeric rSslE there was again evidence of a protein–DNA complex in the well. We then assessed the distribution of SslE and eDNA within established wild-type *E. coli* W biofilms stained with the rSslE antibody and the cell-impermeant DNA-binding dye TOTO-1, respectively, using CSLM (Fig. 7b). Here, we observed TOTO-1 staining across the entire biofilm structure with eDNA localised to dead cells and distributed throughout the biofilm matrix. Labelling of SslE was again seen across biofilms with SslE associated with bacteria, likely through association with the bacterial surface, but also as large clearly defined clusters that co-localised with eDNA and were not visible in the Δ*sslE* control.

## Discussion

In this study, we have revealed that SslE is composed of four defined regions: two globular domains at the N-terminus (NT1 and NT2) and a central torus-shaped core, where a NT3 domain packs against an M60-like aminopeptidase. The N-terminal region (NT1, NT2 and NT3) is composed of a unique primary sequence that has only been identified in SslE and its homologs^41^, while the C-terminal M60-like domain is found in a wide range of proteins secreted by both prokaryotic and eukaryotic microorganisms that interact with mucosal membranes^18,42^. The atomic structures for three M60-like domains in complex with *O*-glycopeptides have been described: BT4244 from *Bacteroides thetaiotaomicron* (PDB ID code: 5kd2), IMPa from *P. aeruginosa* (PDB ID code: 5kdw) and ZmpB from *Clostridium perfringens* (PDB ID code: 5kdn)^19^. While the active site structures in these bacterial enzymes are highly conserved, there are variations within the remaining M60 domain, which enables them to recognise unique glycan sequences. Structural data is not available for intact BT4244 or ZmpB, although they are thought to contain additional carbohydrate-binding domains^18^ and it is likely that these M60 augmentations have a role in the recognition of *O*-glycopeptide substrates. It also appears that the active site in the M60 domain of SslE faces into the NT3 domain and this implies that both the NT3 and M60 domains are responsible for the recognition of mucins in SslE. With significant flexibility of the NT1 and NT2 region, these domains may also interact with mucin substrates and may promote processivity.

Although SslE has a clear function in the processing of mucins, it is a unique member of the M60-like aminopeptidase family as it is also fundamental for the maturation of EPEC biofilms grown under flow. We have also now shown that SslE is required for the maturation of *E. coli* W biofilms, where it is widely distributed throughout the extracellular matrix. We have demonstrated that SslE can form aggregates that display amyloid-like properties at pH ≤ 4.4; conditions which are also observed as microenvironments across mature wild-type *E. coli* W biofilms. It is well established that phenotypical heterogeneity develops during the maturation of biofilms and direct measurement of extracellular pH within *E. coli* PHL628 biofilms has also shown a heterogenous distributions of pH, with values ranging between 5.0 and 7.0^43^. Therefore, it is very likely that SslE can form aggregates within these transiently forming microenvironments and after increases in the local pH they then resist solubilisation. To support this, we have demonstrated that aggregated SslE is deposited throughout macrocolony biofilms and deletion of the *sslE* gene in *E. coli* W and ETEC H10407 resulted in altered colony morphologies.

Microbial amyloid structures are ubiquitous in biofilms and in Gram-negative bacteria most known amyloid structures are synthesised by either the Curli or Fap secretion systems^44–47^. These form through the spontaneous aggregation of extended peptides and provide adhesion, cohesion and contribute to the structural integrity of the biofilm matrix. Furthermore, there are many examples where reduction in pH increases the rate of amyloid formation, through protonation of aspartate and glutamate residues and subsequent structural rearrangements^48,49^. Although our biochemical and biophysical analyses suggest that under acidic condition rSslE displays some amyloid-like features, examination of aggregates using negative-stain TEM has revealed short fibres associated with globular structures on their surface. FTIR analysis of rSslE aggregates provided an amide I maxima at ~1624 cm^−1^, which is consistent with an increase in anti-parallel β-sheet conformation and also lies within the range of amide I maxima for typical amyloid structures (e.g. 1611–1630 cm^−1^)^50^. However, larger, and more rigid amyloid structures generally have peaks at lower frequencies, while native β-sheets show absorption bands with maxima between 1630–1643 cm^−1^. This suggests that the rSslE amide I band peak represents an amyloid structure which is relatively small and is not overly rigid, although it could alternatively originate from fibre stacking/clumping effects. Binding of ThT to rSslE at acidic pH also supports a general increase in β-sheet content in fibres but could also indicate the formation of new cavities upon fibrillation^51^. It is therefore still unclear whether these changes reflect the formation of a continuous amyloid-like structure that runs the length of the fibre core or instead represent β-sheet augmentation between adjacent SslE molecules along the fibre, upon fibrillation of SslE. Nonetheless, based on our biophysical data, we propose a tentative model for the fibrillation of SslE into functional aggregates via end-to-end stacking. In this model, the asymmetric unit of the fibre core is composed of the NT2, NT3 and M60 domains and the NT1 domain is flexible and can move freely around the surface.

Moreover, we have demonstrated that rSslE fibres and monomers can directly bind DNA in vitro and SslE antibodies co-localise with eDNA and bacteria in *E. coli* W biofilms. After secretion SslE can associate with the bacterial membrane but is also present in a soluble form in the extracellular milieu (Supplementary Fig. 7)^4^. Therefore, cell surface-associated SslE could presumably interact with extracellular fibres or may help to nucleate the aggregation of soluble SslE. This could cross-link bacteria and act as a hub to link with eDNA in the matrix. However, it is still unclear which form of SslE is functional in binding eDNA within biofilms and alternatively, monomeric SslE may directly bind eDNA without the need for SslE aggregates. As the nature and consequence of this interaction is not yet clear this now needs more investigation. Nonetheless, we have also presented some evidence that SslE fibres can also associate with cellulose, which is known to have a structural function in biofilms and provides cohesion and elasticity^52^. Although we did not detect cellulose under conditions of SslE production, cellulose secreted by other bacteria in multispecies communities could provide a source for this interaction. As a monomer, SslE does not recognise cellulose, and this suggests that new binding sites are formed after its aggregation. The presence of a surface-exposed domain also implies that this domain may be functional in binding other yet to be identified ligands, or is functional under different conditions, for example through interacting with mucins during intestinal colonisation. Likewise, the fibre core may still retain its mucolytic activity and could play a role in mucin restructuring during biofilm growth in the gut.

Although *E. coli* W, BL21 (DE3), ETEC 10407 and EPEC E2348/69 strains all secrete SslE, the first two strains are harmless commensals, while ETEC and EPEC are major aetiological agents in developing countries^53,54^. SslE is also required for colonisation in a rabbit model of EPEC infection^4^ and is actively transcribed during ETEC infection of mice^6^. This appears to be a common feature of SslE production as expression of *sslE* was previously shown to be repressed at low temperatures in *E. coli* W^5^ and ETEC^7^ and here we have shown that this is also the case in BL21 (DE3) and EPEC E2348/69 strains. This suggests that SslE increases the virulence of *E. coli* pathotypes through its ability to promote biofilm maturation and/or through its interactions with mucosal defences. Both enterotoxigenic and enteropathogenic *E. coli* strains cause infection in the small intestine^1^ where the intraluminal pH ranges between ~6.0 and 7.5^55^. However, *E. coli* possess an acid-tolerance response that supports exponential growth at pH values as low as pH 4.2^56^ and due to the synthesis of organic acids by the residing microbiota, during colonisation of the intestine *E. coli* will experience extracellular pH values that range between 4.0 and 6.0^57^. Together this suggests that SslE is important for infection within the intestinal tract rather than extraintestinal survival, where conditions exist that support its secretion and ability to function as both a monomer and aggregate during biofilm formation. SslE represents a unique protein and further studies are now required to understand its complete functions during ecology and disease.

## Methods

### Media

Lysogeny broth (LB) medium contained (per litre) 10 g tryptone, 5 g yeast extract and 10 g NaCl. LB agar contained (per litre) 10 g tryptone, 5 g yeast extract, 10 g NaCl and 15 g agar. Super optimal broth (SOB) medium essentially contained (per litre) 20 g tryptone, 5 g yeast extract and 0.5 g NaCl. Super Optimal broth with Catabolite repression (SOC) medium was SOC medium with additional 20 mM glucose. YESCA agar contained (per litre) 10 g casamino acids, 1 g yeast extract and 15 g agar. M9-glucose agar contained (per litre) 5.3 g K_2_HPO_4_, 2 g KH_2_PO_4_, 1 g (NH_4_)_2_SO_4_, 0.5 g sodium citrate, 16 g agar, 4 g glucose, 0.1 g MgSO_4_ and 2 μg thiamine. Isotopically defined M9 minimal medium (pH 7.4) contained (per litre) 6.0 g Na_2_HPO_4_·7H_2_O, 3 g KH_2_PO_4_, 0.5 g NaCl, 0.12 g MgSO_4_·7H_2_O, 22 μg CaCl_2_, 40 μg thiamine, 8.3 mg FeCl_3_·6H_2_O, 0.5 mg ZnCl_2_, 0.1 mg CuCl_2_, 0.1 mg CoCl_2_·6H_2_O, 0.1 mg H_3_BO_3_ and 13.5 mg MnCl_2_·6H_2_O, supplemented with 2 g [U-^13^C_6_]glucose and/or 0.7 g ^15^NH_4_Cl. M9 media was made up in deuterium oxide for the production of perdeuterated protein samples and pH was adjusted using 1 M NaOH solution. All NMR isotopes were from Sigma (UK).

### Bacterial strains

All bacterial strains, plasmids and primers used in this study are listed in Supplementary Table 3.

### Gene deletion

A non-polar deletion of *sslE* was constructed in *E. coli* W and H10407 strains by allelic exchange with a FLP recombination target (FRT)-flanked Kan^r^ cassette, using a modified protocol^58^. The fragment was amplified from *E. coli* strain JW5925-1^37^ using Platinum *Taq* DNA polymerase (Promega) and primers PC3 and PC4 and then exchange into *E. coli* W and H10407 strains were facilitated by the λ Red recombinase system carried on plasmid pKD46. *E. coli* strains carrying the pKD46 plasmid were grown in SOB media with 100 μg/ml ampicillin and 1 mM L-arabinose at 30 °C to an OD of 0.6. Cells were made electrocompetent, electroporated with 10–100 ng of PCR fragment and then selected on LB agar at 37 °C containing 25 μg/ml kanamycin, followed by transfer and growth on medium containing no antibiotics. Loss of the pKD46 plasmid was tested by ampicillin sensitivity. Knockouts were confirmed by PCR and sequencing.

### Plasmid construction

Complementation plasmids pCPC1 and pCPC2 were generated by amplifying whole length *sslE*, or *sslE* minus the M60 domain, from *E. coli* W gDNA using primer pairs PC7/PC8 or PC7/PC9, respectively. These were digested with HindIII/NheI (NEB), ligated into HindIII/NheI-digested pBad-cm18 vector. Synthetic genes gPC4, gPC5, gPC6 and gPC7 were synthesised by Synbio Technologies (USA) and cloned into pET28b vector using NcoI and XhoI restriction sites to create plasmids pPC2, pPC3, pPC4 and pPC5, respectively (Supplementary Table 4). Plasmid pBD1 was created by amplification of the RgpB-CTD from *P. gingivalis* W50 gDNA using primers BD1 and BD2. This was then cloned into pET46 Ek/LIC vector through ligation-independent cloning (Novagen).

### Protein purification

Recombinant SslE (rSslE: residues 67–1497; numbered based on mature sequence)^12^, SslE NT1 (residues 67–211), NT2 (residues 230–425), NT1-NT2 (residues 67–425), NT3-M60 (residues 426–1497) and RgpB-CTD were transformed into *E. coli* SHuffle cells (SslE; New England Biolabs) or BL21 (DE3) (RgpB-CTD; New England Biolabs), grown at 37°C in LB media (rSslE, RgpB-CTD: 100 μg/ml ampicillin; NT1, NT2, NT1-NT2, NT3-M60: 100 μg/ml kanamycin) and expression induced with 0.5 mM isopropyl-d-1-thiogalactopyranoside (IPTG) at an OD_600nm_ of 0.6, followed by growth overnight at 18 °C. Cells were resuspended in 20 mM Tris–HCl pH 8, 200 mM NaCl, lysed by sonication and purified using nickel-affinity chromatography followed by gel filtration with either a Superdex 75 (NT1, NT2, RgpB-CTD) or 200 (rSslE, NT1-NT2, NT3-M60) column (GE Healthcare).

### SEC-SAXS

SAXS data were collected on beamline B21^59^ at Diamond Light Source (DLS, Oxford, UK) at 25 °C. 60 μl of rSslE (10 mg/ml) in 20 mM Tris–HCl pH 8, 200 mM NaCl was applied to a KW403-4F column (Shodex) at 0.16 ml/min, preequilibrated in 20 mM citrate-phosphate buffer, 200 mM NaCl at either pH 4.4 or 7.4. SslE N1, N2 and N3-M60 sub-domains were applied to a KW403-4F column (Shodex) at 0.16 ml/min, preequilibrated in 20 mM Tris–HCl pH 8, 200 mM NaCl. SAXS data were measured over a momentum transfer range of 0.003 < *q* < 0.44 Å^–1^. Peak integration and buffer subtraction were performed in CHROMIXS^60^. The radius of gyration (*R*_*g*_) and scattering at zero angle [*I(0)*] were calculated from the analysis of the Guinier region by AUTORG^60^. The distance distribution function [*P(r)*] was subsequently obtained using GNOM^60^ yielding the maximum particle dimension (*D*_max_). Ab initio low-resolution shape restoration was carried out by calculating 20 models in DAMMIF^60^, which were subsequently averaged using DAMAVER^60^ and used as a staring model for refinement in DAMMIN^60^. An additional 20 models were also calculated and averaged in DAMAVER^60^. CRYSOL^60^ was used to compare final rSslE TEM envelopes and models against the solution SAXS curve. Processing and refinement statistics can be found in Supplementary Table 1.

### TEM single-particle analysis

In all, 4 µl of rSslE (625 nM) in 50 mM Tris–HCl pH 8.0, 150 mM NaCl was applied to previously glow-discharged 300 mesh continuous carbon-coated copper grids (Agar Scientific Ltd, UK) for 1 min and blotted for excess liquid. 4 µl of 2% (v/v) uranyl acetate was applied for staining for 1 min. The excess liquid was blotted and left to dry. Data were acquired using a JEOL JEM-2100 plus TEM operating at 200 kV equipped with a OneView 16 Megapixel camera (Gatan). 50 micrographs were collected at a nominal magnification of ×50,000 with a pixel size of 2.1 Å/pixel and a range of defocus from 1 to 3 μm. Data were processed using Relion 3.1^61^. Defocus and astigmatism parameters were estimated using CTFFIND4^62^ in Relion 3.1. An initial dataset of 41,752 particles were autopicked using 2D class averages generated using approx. 1,500 manually picked particles as reference. After a few rounds of 2D classification ignoring CTF until the first peak, 10,988 particles were taken forward for an initial model generation using 3D initial model in Relion 3.1^61^. Following initial model generation, a few rounds of 3D classification followed by 3D refinement was carried out. The final model was refined to 22 Å using the gold standard FSC (0.143 criterion). PHYRE2^63^ was used to analyse the SslE sequence (residues 67–1497; UniProt ID E0IW31) and generate a homology model for the C-terminal M60 domain (residues 962–1415) based on residues 421–894 of *P. aueriginosa* IMPa (PDB ID code 5kdv; 100% confidence, 21% identity)^19^. This was then docked into the rSslE TEM envelope using UCSF CHIMERA^64^.

### Solution NMR spectroscopy

NMR measurements for rSslE were performed at 25 °C on a 100 µM ^2^H^15^N-labelled sample in 50 mM NaPO_4_ pH 7.4, 100 mM NaCl, 10 % D_2_O. NMR measurements for SslE NT1 (0.6 mM), SslE NT2 (1.3 mM) and SslE NT1-NT2 (0.8 mM) were performed at 25 °C on ^15^N-labelled samples in 50 mM NaPO_4_ pH 7.0, 100 mM NaCl, 10 % D_2_O. NMR measurements for RgpB-CTD (0.3 mM) were performed at 37 °C on a ^15^N-labelled sample in 20 mM NaPO_4_ pH 6.0, 100 mM NaCl, 10% D_2_O. Transverse relaxation optimised spectroscopy (TROSY) based ^1^H^15^N-HSQC experiment and *T*_1_ and *T*_2_ relaxation times for rSslE were recorded on a Bruker Avance III HD 950, equipped with a TXI cryoprobe. TROSY ^1^H^15^N-HSQC experiment for SslE NT1-NT2 was recorded on a Bruker Avance III HD 800, equipped with a TCI cryoprobe. Standard ^1^H^15^N-HSQC spectra of SslE NT1, SslE NT2 and RgpB-CTD were recorded on a Bruker Avance III HD 700, equipped with a TCI cryoprobe. Data were processed in NMRPIPE^65^ and analysed/visualised with ANALYSIS^66^ and NMRVIEW^67^.

### Recombinant protein ring assay

In all, 20 µl of rSslE, NT1-NT2 or NT3-M60 (62.5 µM) in 10 mM Tris–HCl pH 8, 100 mM NaCl buffer were diluted to 2 ml in 100 mM citrate-phosphate buffer at pH 4.0, 4.2, 4.4, 4.6, 4.8, 5.0, 5.4, 5.8, 6.2, 6.6 and 7.0. These were transferred to glass tubes, incubated overnight at 37 °C while shaking (200 rpm) and the presence of ring formation on the glass tube was visually assessed. Buffer from tubes containing rings formed at pH 4.0 was then removed without disturbing the rings and 3 ml of 100 mM citrate-phosphate buffer over the same pH range was added. These were left overnight with shaking at 37 °C and solubilisation of the rings were visually assessed the next day.

### Mass spectrometry

Ring aggregates formed from either bacterial cultures or recombinant SslE were scraped from glass tubes and resuspended in 0.5 ml 100 mM citrate-phosphate buffer, pH 4.0. Samples were resolved using SDS PAGE and after staining, SslE fibre bands retained in the wells were excised and incubated with 10 mM dithiothreitol at 56 °C and then alkylated with 55 mM iodoacetamide at room temperature. Samples were digested using 1:20 dilution of bovine trypsin incubated in a shaking heat block at 37 °C for 16 h. Peptides were extracted with aqueous dehydration/hydration using acetonitrile and 50 mM triethylammonium bicarbonate, pooled and dried. Samples were resuspended in 2% (v/v) acetonitrile, 0.05% (v/v) formic acid and peptides were resolved by reversed-phase chromatography on a 75 μm C18 Pepmap column (50 cm length) using a three-step linear gradient of 80% acetonitrile in 0.1% formic acid (U3000 UHPLC NanoLC system; ThermoFisherScientific, UK). The gradient was delivered to elute the peptides at a flow rate of 250 nl/min over 60 min starting at 5% B (0–5 min) and increasing solvent to 40% B (5–40 min) prior to a wash step at 99% B (40–45 min) followed by an equilibration step at 5% B (45–60 min). The eluate was ionised by electrospray ionisation using an Orbitrap Fusion Lumos (ThermoFisherScientific, UK) operating under Xcalibur v4.1.5. The instrument was first programmed to acquire using an Orbitrap-Ion Trap method by defining a 3 s cycle time between a full MS scan and MS/MS fragmentation. Orbitrap spectra (FTMS1) were collected at a resolution of 120,000 over a scan range of m/z 375–1500 with an automatic gain control (AGC) setting of 4 × 10^5^ with a maximum injection time of 35 ms. Monoisotopic precursor ions were filtered using charge state (+2 to +7) with an intensity threshold set between 5 × 10^3^ and 1 × 10^20^ and a dynamic exclusion window of 35 secs ± 10 ppm. MS2 precursor ions were isolated in the quadrupole set to a mass width filter of 1.6 *m/z*. Ion trap fragmentation spectra (ITMS2) were collected with an AGC target setting of 1 × 10^4^ with a maximum injection time of 35 ms with CID collision energy set at 35%. Data were processed using Proteome Discoverer (v2.2; ThermoFisher) to search against Uniprot Swissprot All Taxonomy (561,911 entries) and the sequence of SslE (Uniprot Accession number—E3PJ90) with Mascot search algorithm (v2.6.0; www.matrixscience.com) and the Sequest search algorithm^68^. Precursor mass tolerance was set to 20 ppm with fragment mass tolerance set to 0.8 Da with a maximum of two missed cleavages. Variable modifications included: Carbamidomethylation (Cys) and Oxidation (Met). Database generated files (.msf) were uploaded into Scaffold software (v 4.10.0; www.proteomesoftware.com) for visualisation of fragmentation spectra.

### ThT binding

In all, 15 µl of 50 µM rSslE in 10 mM Tris–HCl pH 8, 100 mM NaCl buffer was mixed with 1.5 µl of 1 mM Thioflavin-T (ThT) dye and transferred to a 96-well plate. 100 mM citrate-phosphate buffer between pH 4.0 and 8.0 was added to a final volume of 150 µl and fluorescence data were collected (excitation/emission 438/480 nm) at 37 °C with shaking every 15 min over 24 h using a BMG CLARIOstar plate reader.

### SslE fibre immunoblot

Ring aggregates formed from rSslE were scraped from glass tubes and resuspended in 0.5 ml 100 mM citrate-phosphate buffer, pH 4.0, centrifuged at 15,000 × *g* and then the top 950 µl solution was carefully removed and discarded. This was followed by three rounds of addition of 950 µl 100 mM citrate-phosphate buffer at pH 4.0, centrifugation at 15,000 × *g* and the top 950 µl discarded. The final 50 µl sample was mixed with 1 × NuPAGE LDS Sample Buffer (ThermoFisher), 5% (v/v) β-mercaptoethanol and incubated at 100 °C for 5 min prior loading. This was run on a Criterion 4–20% SDS-PAGE gel (Bio-Rad), followed by transfer onto a PVDF membrane using the semi-dry Invitrogen Power–Blotter and Power–Blotter transfer blotting solution. The membrane was blocked in 1% (w/v) BSA, PBS-Tween for 1 h at room temperature followed by the addition of 1:2000 dilution mouse anti-His_6_ antibody (Sigma) in 0.5% (w/v/) BSA, PBS-Tween incubation buffer for 2 h. After five rounds of washing with incubation buffer, the membrane was incubated with 1:2000 anti-mouse HRP-conjugated antibody (Sigma) for 1 h, followed by five further washes and then treatment with ELC substrate (Peirce) before detection. Raw immunoblots are provided in Supplementary Fig. 23a.

### Far-UV CD spectroscopy

Far-UV CD spectra were measured on a Chirascan (Applied Photophysics) spectropolarimeter thermostated at 25 °C. rSslE (10 mg/ml) in 20 mM Tris–HCl pH 8, 50 mM NaCl was diluted 200-fold to 0.05 mg/ml in 10 mM MOPS at either pH 4.4 or 7.4. Spectra were recorded from 260 to 190 nm, at 0.2 nm intervals, 1 nm bandwidth, and a scan speed of 50 nm/min. Three accumulations were averaged for each spectrum. Deconvolution of data was performed using the BESTSEL server^69^.

### ATR FT-MIR spectroscopy

FT-MIR spectroscopy measurements were acquired using a Perkin Elmer Frontier spectrometer (Perkin Elmer, Buckinghamshire, UK) in attenuated total reflectance (ATR) mode, fitted with a Golden Gate ATR accessory (Specac Ltd., Kent, UK) with a diamond crystal. Samples were enclosed in a sealed Plexiglas container within a nitrogen-filled atmosphere at 23 ± 1 °C with additional calcium chloride dihydrate pellets (Sigma). Spectra were acquired using an infrared light source and a DTGS (deuterated triglycine sulphate) detector over a spectral range of 500–4000 cm^−1^ with a 4 cm^−1^ spectral resolution (filling factor = 2) and 64 co-added scans. 5 µl rSslE solution, as either soluble monomer in PBS buffer (pH 7.4) or aggregates in 100 mM phosphate-citrate buffer (pH 4.4), was deposited and dried onto the ATR crystal (*n* = 3 per protein solution). Spectral data were background-subtracted and baseline corrected using the Spectrum One software package (version 6, Perkin Elmer, Buckinghamshire, UK).

### Bacterial culture ring assay

In total, 10 ml of LB containing 0.8 mg/ml Congo red dye, adjusted to either pH 5.0 or 7.0 with HCl, were inoculated with wild-type *E. coli* W and derivatives (including 50 µg/ml kanamycin for *Δssle* mutant and 50 µg/ml kanamycin, 25 µg/ml chloramphenicol for *Δssle::pCPC1* (*sslE*) and *Δssle::pCPC2* (*sslEΔM60*)). These were incubated overnight at 37 °C while shaking (200 rpm) and the presence of ring formation on the glass tube was visually assessed.

### SslE secretion

*E. coli* W strains were grown on LB agar with 50 µg/ml kanamycin for *Δssle* mutants and 50 µg/ml kanamycin, 25 µg/ml chloramphenicol for *Δssle::pCPC1* (*sslE*) and *Δssle::pCPC2* (*sslEΔM60*). Single colonies were resuspended in 10 ml LB (with appropriate antibiotics) and incubated overnight at 37 °C with shaking (200 rpm). Cells were centrifuged to separate out the media (supernatant) and pellet (whole cells) and 3 µl of samples were resuspended in 17 µl of 1× NuPAGE LDS Sample Buffer (ThermoFisher), 5% (v/v) β-mercaptoethanol. *Samples were* run on a Criterion 4–20% SDS-PAGE gel (Bio-Rad), followed by transfer onto a PVDF membrane using the semi-dry Invitrogen Power–Blotter and Power–Blotter transfer blotting solution. The membrane was blocked with 1% (w/v) BSA, PBS-Tween for 1 h at room temperature, and then incubated overnight at 4 °C with polyclonal anti-rSslE antibody (rabbit; Invitrogen) or monoclonal anti-DsbA (mouse; Invitrogen), diluted 1:1000 using 0.5% (w/v) BSA, PBS-Tween incubation buffer. After three, 5 min washes with incubation buffer, membranes were incubated for 1 h at 37 °C with either anti-rabbit or anti-mouse secondary antibody conjugated to HRP (1:2000 dilution; Invitrogen) for 1 h at 25 °C and then treated with enhanced chemiluminescence substrate (ECL; Pierce) before detection.

### Immunoelectron microscopy

Overnight cultures were incubated for 30 min in 20 mM citrate-phosphate buffer at either pH 4.0 or 5.0 and then washed in the same buffer before fixing with the 3% (w/v) paraformaldehyde for 1 h. Cells were then loaded onto a Glow-discharged carbon-coated Ni grid (Agar Scientific) for 10 min, washed with 50 mM glycine and then blocked with 1% (v/v) Natural Donkey Serum (Jackson Immunoresearch) for 30 min. This was then incubated for 1 h with either primary polyclonal rSslE antibody (rabbit; Invitrogen) or primary polyclonal CsgA antibody (guineapig; Invitrogen) diluted 1:100 in blocking buffer, washed five times for 3 min each with 0.05% Natural Donkey Serum and then incubated for 1 h with gold-conjugated secondary antibody (donkey; Jackson Immunoresearch) diluted 1:100 in blocking buffer. Washing was repeated and the samples were negatively stained with 2% (w/v) uranyl acetate for 30 s, followed by two quick washes with ddH_2_O. The grid was air-dried, and images were recorded on a JEM-1230 (JEOL-Japan) at 80 kV with a Morada CCD camera, iTEM software (EMSIS). Images of 13 *E. coli* W wild-type and 13 *Δssle* mutant bacteria were then masked and gold particles were counted between ~0.2 and 1.0 µm from the bacterium.

### TEM fibre analysis

rSslE at 1 mg/ml was incubated in 100 mM citrate-phosphate pH 3.8 overnight at room temperature while shaking at 180 rpm. This was centrifuged at 15,000 × *g* for 10 min, the top 80% of buffer discarded and then 4 µl of the remaining sample was applied to a previously glow-discharged 300 mesh continuous carbon-coated copper grids (Agar Scientific Ltd) and immediately blotted for excess liquid. In all, 4 µl of 2% (v/v) uranyl acetate was applied for staining for 10 s. The excess liquid was blotted and left to dry. Data were acquired using a JEOL JEM-1230 TEM operating at 80 kV equipped with a Morada 2k CCD camera system and its iTEM software package (Olympus Europa, UK). Micrographs were collected at a nominal magnification of 80,000x with a pixel size of 5.96 Å/pixel.

### RT-MALS

In total, 1 ml of rSslE (1 mg/ml) in 25 mM citrate-phosphate buffer pH 4.0 was incubated overnight at room temperature while shaking at 180 rpm. The sample was then centrifuged at 15,000 × *g* and the top 850 µl of buffer was discarded. MALS experiments were then performed on beamline B21 at the DLS (Oxford, UK). In total, 20 µL of SslE fibres were directly injected into the RT-MALS multiple times at a flow rate of 0.05 ml/min. Detectors were standardised through a direct injection using BSA. Data were analysed using ASTRA version 6.1.7. (Wyatt).

### Solid-state NMR spectroscopy

rSslE at 20 mg/ml was buffer exchanged into 10 mM citrate-phosphate pH 4.0 using a PD10 column (Sigma) and then incubated overnight at room temperature while shaking at 180 rpm. This was centrifuged at 15,000 × *g* for 10 min, the top 80% of buffer was discarded and then the remaining sample was used for solid-state NMR analysis. Experiments were performed using a Bruker Neo Console operating at 850 MHz ^1^H frequency with a 3.2 mm E-Free probe in HC mode spinning at a rate w_r_ = 15 kHz. A standard CP excitation ^13^C-^13^C DARR experiment was acquired. The direct dimension was acquired for 16.4 ms with ~55 kHz SPINAL-64 decoupling. 256 rows with 256 co-added transients and a recovery delay of 2.5 s were acquired using Time-Proportional Phase Increment (TPPI) with a 22.2 µs dwell (45 kHz sweep width, 2.84 ms total evolution) for a total of 45.5 h total acquisition time. The applied power was adjusted so that ^1^H and ^13^C hard pulses were both 4 µs (w_1(H,C)_ = 62.5 kHz). The initial carbon excitation was achieved with 1.5 ms of ramped Hartmann–Hahn CP, where the optimal polarisation transfer was found at w_1C_ = ~70 kHz and w_1H_ = ~55 kHz with an upwards linear ramp from 70–100% on the ^1^H channel. Homonuclear Carbon mixing was achieved with 50 ms of DARR mixing (w_1H_ = w_r_ = 15 kHz).

### SAXS fibrillation analysis

SAXS data were collected on beamline B21^59^ at DLS (Oxford, UK) at 25 °C. Immediately prior to data collection, 10 mg/ml rSslE in 20 mM Tris–HCl pH 8, 200 mM NaCl was buffer exchanged into 20 mM citrate-phosphate buffer, pH 4.4 using a PD10 column (GE Healthcare) and the flow-through was used as a buffer reference. Using a peristaltic pump and while constantly stirring, 5 ml of rSslE (0.7 mg/ml) was circulated through the SAXS imaging cuvette and data were collected every 30 min over a momentum transfer range of 0.004 < *q* < 0.4 Å^−1^, with the initial scattering data captured at 30 min after initiating fibre growth. Data collected over the course of 11 h, consisting of 22 profiles, was decomposed using COSMiCS^30^, which utilises MCR-ALS^70^ to perform model-free decomposition of the entire SAXS dataset. The SEC-SAXS curve collected at pH 4.4 was also included in the dataset as the representative state of the protein at time 0 s, i.e. before initiating fibrillation. The time 0 s curve was selected as one of the initial estimates and the selectivity restraint was used to ascertain that the curve had no contribution from the other species. In addition, non-negativity restraint was imposed for both the SAXS profiles and concentrations using FNNLS algorithm^71^. Before COSMiCS analysis, SAXS data were scaled according to the large angle data to enhance the decomposition capacity of the approach. Although COSMiCS was run assuming a two-component system as suggested by Principal Component Analysis (PCA), a three-species run was also performed. The convergence criterion of <0.01% change in lack of fit was used with 1000 maximum allowed iterations. The analysis of the resulting COSMiCS curves was performed with the ATSAS suit of programmes^60^. Fractal analysis was carried out using SASVIEW version 4.2.2 (http://www.sasview.org/). The programmes ATSAS^60^ and Scatter were also used to obtain a cross-sectional radius of gyration *R*_*c*_ from the 10 h post-fibre induction scattering profile and from this the cross-sectional *P*(*r*)_*C*_ was calculated. BODIES^60^ was then used to approximate the geometric shape of rSslE fibres, which suggested a cylindrical shape with radius of 3.93 nm and a height of 16.5 nm (χ^2^ 1.6). Processing and refinement statistics can be found in Supplementary Table 1.

### Macrocolony biofilm growth

In all, 5 µl of wild-type *E. coli* W, BL21 (DE3), H10407 and E2348/69 strains and their derivatives were grown on LB agar, YESCA agar or M9-glucose agar containing either 0.8 mg/ml Congo red dye or 50 µM Fluorescent Brightener 28 (Sigma, UK) at 37 °C, 25 °C or 18 °C for either 24 or 96 h. In total, 50 µg/ml kanamycin was included for W and H10407 Δ*ssle*, E2348/69 Δ*csgA*, BW25113 pgaC and BW25113 wcaF mutant strains, and 50 µg/ml kanamycin, 25 µg/ml chloramphenicol was included for the E2348/69 Δ*csgA*/Δ*bcsA* mutant and W *Δssle::pCPC1* (*sslE*) and *Δssle::pCPC2* (*sslEΔM60*).

### Microfluidic biofilm growth

Biofilms were grown in a BioFlux 200 device (Labtech, UK) using a protocol adapted from the manufacturer. Single colonies of *E. coli* W and its derivatives were resuspended in 10 ml of LB (with additional 50 µg/ml kanamycin for *Δssle* mutant and 50 µg/ml kanamycin, 25 µg/ml chloramphenicol *Δssle::pCPC1* (*sslE*) and *Δssle::pCPC2* (*sslEΔM60*)) and incubated at 37 °C with shaking (200 rpm) for 16 h. All subsequent media contained appropriate antibiotics where required. The cultures were then diluted (1:100) in 10 ml of LB to OD_600nm_ of 0.05. A 24-well Bioflux plate (0–20 dyn/cm^2^) was prewarmed to 37 °C on a heated stage and microfluidic channels were incubated with prewarmed LB. The channels were then inoculated by injecting 20 µl of bacterial suspension into the output reservoir for 5 s at 2 dyne/cm^2^. The microfluidic plate was incubated for 1 h at 37 °C to allow bacteria to bind to the surface and then the flow reversed for 20 s. Prewarmed LB was then added to the input reservoir, the flow of media was initiated at 2 dyne/cm^2^ for 5 min and then decreased to 1 dyne/cm^2^ for 18 h. The spent media was removed from the output reservoir, fresh prewarmed media was added to the input reservoir and the flow was lowered to 0.5 dyne/cm^2^ for up to 96 h.

### Assessment of biofilm growth

FilmTracer FM 1–43 dye (Life Technologies) was added to the BioFlux 200 input reservoir mixed with prewarmed LB to a final concentration of 0.3 µM. The heated stage holding the BioFlux plate was then moved to a DM-IRE2 confocal laser scanning microscope (Leica Microsystems Heidelberg GmbH, Germany) for image acquisition with the Leica Microsystems Confocal Software (version 2.61 Build 1537). The fluorophore was excited at 488 nm and emission collected at 580 nm as recommended by the manufacturer.

### Biofilm pH measurement

The ratiometric dye seminaphthorhodafluor-4F 5-(and-6) carboxylic acid (C-SNARF-4) was used to directly measure pH across biofilms within the Bioflux channel, using a modified method^72^. HEPES buffer was first adjusted between pH 3.2 and 7.8 in 0.2 increments using HCl, and 95 μl of each respective solution was then mixed with 5 μl 1 mM C-SNARF-4, resulting in a final 50 μM concentration of C-SNARF-4. This solution was flushed into the Bioflux plate channel, prewarmed at 37 °C and the channels were imaged at ×64 magnification using a DM-IRE2 confocal laser scanning microscope (Leica Microsystems Heidelberg GmbH, Germany) and the accompanying Leica Microsystems Confocal Software (version 2.61 Build 1537). Simultaneous images were captured using an excitation wavelength of 488 nm and emission wavelengths of 580 nm and 640 nm, in triplicate for each pH increment with a background image obtained after each acquisition. Acquired images were analysed using ImageJ Software (FIJI)^73^ by determining the average intensity of fluorescence and standard deviation of each channel, minus the background control. The intensity ratio for each pH was used to make a standard curve by plotting known pH against green/red ratio. 50μM C-SNARF-4 was then flushed into Bioflux plates containing *E. coli* W biofilm after either 24 or 96 h growth and five separate images were acquired for each Bioflux channel to calculate the pH from different areas of the biofilm. The fluorescence intensity and standard deviation were recorded, and the ratiometric value was used to determine pH from the standard curve. A 12 × 12 grid (∼30 μm^2^ area) was applied to each image; each box was used to define regions of interest (ROI) and an average measure of pH was calculated within each region. The average pH for ROIs within the biofilm fringes and centres is presented.

### Cellulose quantification

A lawn of wild-type *E. coli* W, BL21 (DE3), H10407 and E2348/69 strains or their derivatives were scraped from the surface of LB agar or YESCA agar plates after 96 h at 37 °C or 25 °C. For each strain, ~30 mg of cells was transferred into 400 µl of 2.5 M 2-(N-Morpholino)-ethanesulfonic acid (MES) buffer pH 5.5 with or without 6 U/ml cellulase (Sigma, UK) and incubated at 37 °C overnight. Each sample was adjusted to a final turbidity of 40 at 600 nm with 2.5 mM MES pH 5.5 and then centrifuged at 5000×*g* for 20 min. The glucose concentration in each sample was measured using the glucose (HK) assay kit (Sigma, UK) and compared with data from none cellulase treated samples.

### Curli and SslE immunoblot

A lawn of wild-type *E. coli* W, BL21 (DE3), H10407 and E2348/69 strains or their derivatives were scraped from the surface of LB agar or YESCA agar plates after 96 h at 37 °C or 25 °C and transferred to 1 ml PBS and the volume adjusted to an OD_600nm_ = 1.0. Formic acid was added to 70% (v/v) and then lyophilised overnight. Samples were resuspended in 200 µl 1× NuPAGE LDS loading buffer (ThermoFisher) and run on a Criterion 4–20% SDS-PAGE gel (Bio-Rad), followed by transfer onto a PVDF membrane using the semi-dry Invitrogen Power–Blotter and Power–Blotter transfer blotting solution. The membrane was blocked with 3% (w/v) BSA, PBS-Tween for 1 h at room temperature followed by the addition of 1:1000 dilution polyclonal primary anti-rSslE antibody (rabbit; Invitrogen) or 1:5000 dilution polyclonal anti-CsgA antibody (guineapig; Invitrogen) in 0.5% (w/v/) BSA, PBS-Tween incubation buffer for 1 h. After 3 rounds of washing with incubation buffer, the membrane was incubated with 1:2000 HRP-conjugated anti-rabbit or anti-guineapig antibody (Sigma), respectively, for 1 h, followed by three further washes and then treatment with ELC substrate (Peirce) before detection. Raw immunoblots are provided in Supplementary Fig. 23b, c.

### PNAG dot–blot and overlay assay

A lawn of wild-type *E. coli* W, BL21 (DE3), H10407, E2348/69 and BW25113 strains or their derivatives were scraped from the surface of LB agar plates after 96 h at 37 °C or 25 °C. For each strain, bacteria were transferred into 3 ml of 0.5 M EDTA (pH 8.0) per g of cells, followed by 5 min incubation at 100 °C. Samples were centrifuged at 10,500 × *g* for 6 min and then 100 μl of supernatant was incubated with 10 μl of proteinase K (20 mg/ml; NEB, UK) for 60 min at 60 °C, followed by 80 °C for 30 min. This was diluted threefold with ddH_2_0 and then spotted onto a PVDF membrane. The membrane was dried, blocked with 3% (w/v) BSA, PBS-Tween for 1 h at room temperature and then incubated with 70 μg/ml wheat germ agglutinin Biotin-conjugated (WGA-biotin; Sigma, UK) in 0.5% (w/v) BSA, PBS-Tween for 1 h at room temperature. After four rounds of washing with incubation buffer, the membrane was incubated with 1:2000 HRP-conjugated streptavidin (Sigma, UK) for 1 h, followed by 3 further washes and then treatment with ELC substrate (Peirce) before detection. For overlay assays membranes were blocked with 3% (w/v) BSA in 100 mM citrate-phosphate buffer pH 6.0, 0.05% (w/v) Tween-20 and then incubated with either 30 µM rSslE monomer, rSslE fibre (produced as described for immunoblotting) or RgpB-CTD control, all in 0.5% (w/v) BSA, 100 mM citrate-phosphate buffer pH 6.0, 0.05% (w/v) Tween-20, for 1 h at room temperature. After four rounds of washing with 0.5% (w/v) BSA, 100 mM citrate-phosphate buffer pH 6.0, 0.05% (w/v) Tween-20, the membrane was incubated with 1:2000 anti-His_6_ HRP-conjugated antibody (ThermoFisher, UK) in the same buffer for 1 h, followed by 4 further washes and treatment with ELC substrate (Peirce) before detection.

### Colanic acid quantification

A lawn of wild-type *E. coli* W, BL21 (DE3), H10407, E2348/69 and BW25113 strains or their derivatives were scraped from the surface of LB agar or M9-glucose agar plates after 96 h at 37 °C, 25 °C or 18 °C and resuspended in ddH_2_0 to a final OD_600nm_ = 1.0. After incubating at 100 °C for 10 min and centrifuging at 16,000 × *g* for 10 min, 100 µl of supernatant was then diluted to 1 ml with ddH_2_0 and mixed with 4.5 ml H_2_SO_4_/H_2_O (6:1; v/v). This was boiled at 100 °C for 20 min, cooled to room temperature, divided in two and then 50 µl ddH_2_O (control) was added to one sample and 50 μl 1.0 M cysteine hydrochloride (Sigma, UK) added to the other sample. Absorbance was measured at 396 nm and 427 nm and then control subtracted from the cysteine hydrochloride values. A standard curve of l-fucose (Sigma, UK) was produced using concentrations ranging from 5 to 100 μg/ml plotted against absorbance at 396 nm and 427 nm. This was used to determine the amount of fucose released from colanic acid samples, which were then scaled and normalised based on the cell density measurements.

### Cellulose-binding assay

An Immulon 2-HB 96-well plate (VWR) was blocked for 1 h at 25 °C with 300 µl of 0.1% (w/v) BSA in PBS-Tween and then washed once with 300 μl of incubation buffer (100 mM citrate-phosphate buffer pH 6.0, 0.05% (w/v) BSA, 0.05% Tween-20). One *Komagataeibacter rhaeticus* cellulose disc^74^ was added to each well and then 200 μl of incubation buffer was added to cover the discs, followed by incubation for 5 min. The discs were washed twice in incubation buffer and then 200 µl of either 100 µM rSslE monomer, rSslE fibre (produced as described for immunoblotting) or RgpB-CTD control, all in incubation buffer, were added to the cellulose discs. The plate was incubated for 3 h at 25 °C and then the discs were transferred carefully to new pre-blocked wells and washed with 200 μl incubation buffer. After repeating three times, the discs were incubated with anti-His_6_ HRP-conjugated antibody (ThermoFisher) diluted 1:2000 with incubation buffer for 1 h at 24 °C. The discs were removed again, and the washing step was repeated three times. Finally, the solution was removed from the wells and the discs were incubated with 150 μl of *o*-Phenylenediamine dihydrochloride (Sigma) for 30 min. The solution in the wells was stirred thoroughly, the discs were removed and then data were recorded at 450 nm.

### Electrophoretic mobility shift assay

In all, 10 nM of pUC19 plasmid DNA (NEB, UK) was mixed with 0, 12.5, 25, 50 and 100 µM of either rSslE monomer, rSslE fibre (produced as described for immunoblotting) or RgpB-CTD control in 10 mM 2-(N-morpholino) ethanesulfonic acid (MES) pH 6.0, 50 mM NaCl, 10% (v/v/) glycerol, and incubated for 1 h at room temperature. The DNA-fibre sample was then mixed with 60% glycerol, 0.01% (v/v) bromophenol blue in MES buffer pH 6.0, while DNA-monomeric rSslE and RgpB-CTD samples were mixed with “purple no SDS DNA loading dye” at pH 8.0 (NEB, UK). Samples were loaded onto a 1% (w/v) agarose gel containing GelRed (Biotium, UK) in TBE buffer and run at 70 V for 45 min at 4 °C and visualised using UV light.

### Biofilm immunofluorescence

Single colonies of wild-type *E. coli* W and *Δssle* mutant control were resuspended in 10 ml of LB and incubated at 37 °C with shaking (200 rpm) for 16 h. Cultures were diluted (1:100) in prewarmed LB and 1 ml was injected onto borosilicate glass coupons held within FC310 flow cells (Biosurface Technology) and incubated for 1 h. The flow of LB media was initiated at 0.25 ml/min and maintained for 96 h. Coupons were removed and washed three times for 5 min each in PBS. Coupons were then blocked for 1 h in 2% (w/v) BSA, PBS at room temperature and incubated overnight at 4 °C with 10 μg/ml polyclonal anti-rSslE antibody (rabbit; Invitrogen) in 0.1% (w/v) BSA, PBS. After three 5 min washes in PBS, coupons were incubated in the dark for 1 h with either 1 μM TOTO-1 (Invitrogen) or 1 μg/ml FM1-43 (Invitrogen), and anti-rabbit Alexa Fluor 633 secondary antibody (goat; Invitrogen) diluted 1:500 in 0.1% (w/v) BSA, PBS. This was followed by three 5 min washes in the dark in PBS and then images were captured at ×20 magnification using an excitation wavelength of 488 nm (TOTO-1), 488 nm (FM1-43), and 633 nm (Alexa Fluor 633), and emission wavelengths of 534 nm, 581 nm and 650 nm, respectively, with a DM-IRE2 confocal laser scanning microscope (Leica Microsystems Heidelberg GmbH, Germany). Additional negative controls consisted of *E. coli* W biofilms being incubated with either no primary anti-rSslE antibody or no Alexa Fluor 633 secondary antibody.

### Reporting summary

Further information on research design is available in the Nature Research Reporting Summary linked to this article.

## Supplementary information

SUPPLEMENTAL MATERIALS

Reporting Summary

## Data Availability

The data that support the findings of this study are included in the article, its supplementary information files, or are available from the corresponding author upon reasonable request. The plasmids pPC1, pPC2, pPC3, pPC4, pPC5, pBD1, pCPC1 and pCPC2 have been deposited in the Addgene plasmid repository (https://www.addgene.org) with IDs 182013, 182014, 182015, 182016, 182017, 182018, 182019 and 182020, respectively. The negative-stain TEM electron density map for SslE pH 7.4 has been deposited in the Electron Microscope Data Bank (https://www.ebi.ac.uk/emdb) with accession code EMD-14170. The buffer subtracted SAXS curves and DAMMIN models with the lowest NSD score for monomeric SslE at pH 4.4 and 7.4, and the SslE N1, N2 and N3-M60 domains, have been deposited in the Small-Angle Scattering Biological Data Bank (https://www.sasbdb.org/) with accession ID SASDLW2, SASDLV2, SASDMU6, SASDMV6 and SASDMW6, respectively.
