# Supplementary material for: Molecular and cellular insight into Escherichia coli SslE and its role during biofilm maturation
Source: NPJ Biofilms Microbiomes. 2022 Feb 25;8:9. doi: 10.1038/s41522-022-00272-5 (PMC8881592; doi:10.1038/s41522-022-00272-5)
Supplement: Supplementary file 1 — SUPPLEMENTAL MATERIALS [file 41522_2022_272_MOESM1_ESM.pdf]

Supplementary Information

Molecular and cellular insight into *Escherichia coli* SslE and its role during biofilm maturation

Paula M. Corsini[1,2], Sunjun Wang[1], Saima Rehman[1], Katherine Fenn[2], Amin Sagar[3], Slobodan Sirovica[4], Leanne Cleaver[1], Charlotte J. C. Edwards-Gayle[5], Giulia Mastroianni[2], Ben Dorgan[1], Lee M. Sewell[1], Steven Lynham[6], Dinu Iuga[7], W. Trent Franks[7], James Jarvis[8], Guy H. Carpenter[1], Michael. A. Curtis[1], Pau Bernadó[3], Vidya C. Darbari[2] & James A. Garnett[1,2]

[1]*Centre for Host-Microbiome Interactions, Faculty of Dental, Oral & Craniofacial Sciences, King's College London, London, UK*

[2]*School of Biological and Behavioural Sciences, Queen Mary University of London, London, UK*

[3]*Centre de Biologie Structurale. INSERM, CNRS, Université de Montpellier, France*

[4]*Centre for Oral Bioengineering, Institute of Dentistry, Barts and The London School of Medicine and Dentistry, Queen Mary University of London, London, UK*

[5]*Diamond Light Source Ltd., Diamond House, Harwell Science and Innovation Campus, Didcot, Oxfordshire, UK*

[6]*Proteomics Facility, Centre of Excellence for Mass Spectrometry, King's College London, London, UK*

[7]*Department of Physics, University of Warwick, Coventry, UK*

[8]*Randall Division of Cell and Molecular Biophysics and Centre for Biomolecular Spectroscopy, King's College London, London, UK*

Supplementary Figures

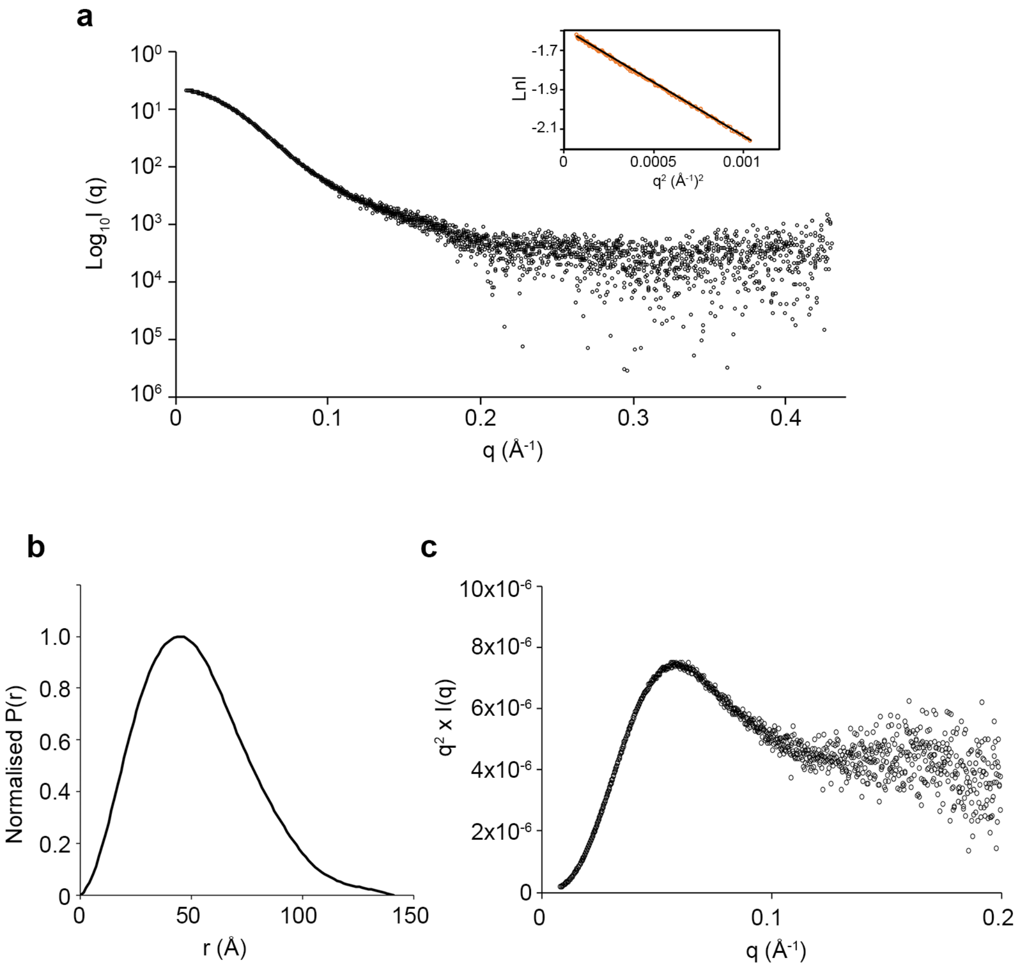

Supplementary Fig. 1 | SAXS analysis of rSslE at pH 7.4. a, Experimental scattering curve of rSslE (black open circles). Inset: Guinier Region (orange open circles) and linear regression (black line) for R_g evaluation. **b,** Shape distribution (*P(r)*) function derived from SAXS analysis for rSslE. **c,** Kratky plot indicates that rSslE has dynamic features in solution.

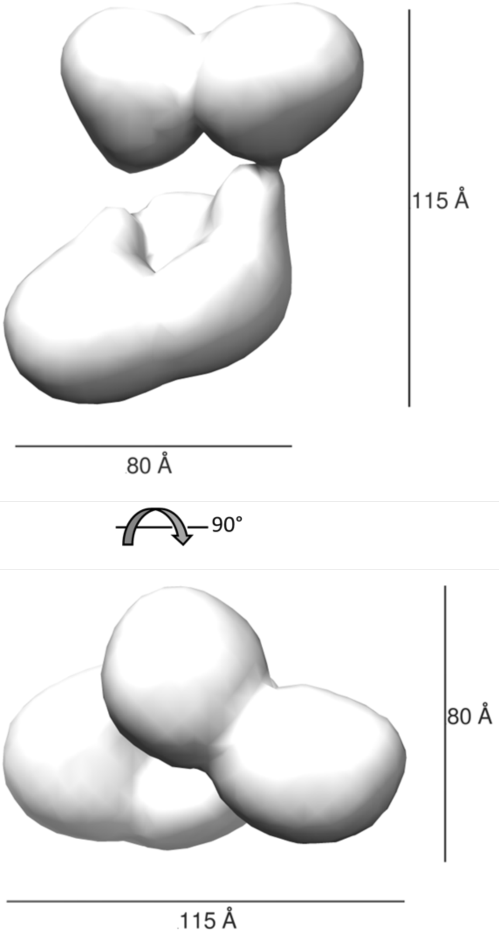

Supplementary Fig. 2 | Overall dimensions of rSslE. Negative-stain TEM map of rSslE is shown in two orientations with overall dimension of ~8×8×11.5 nm annotated.

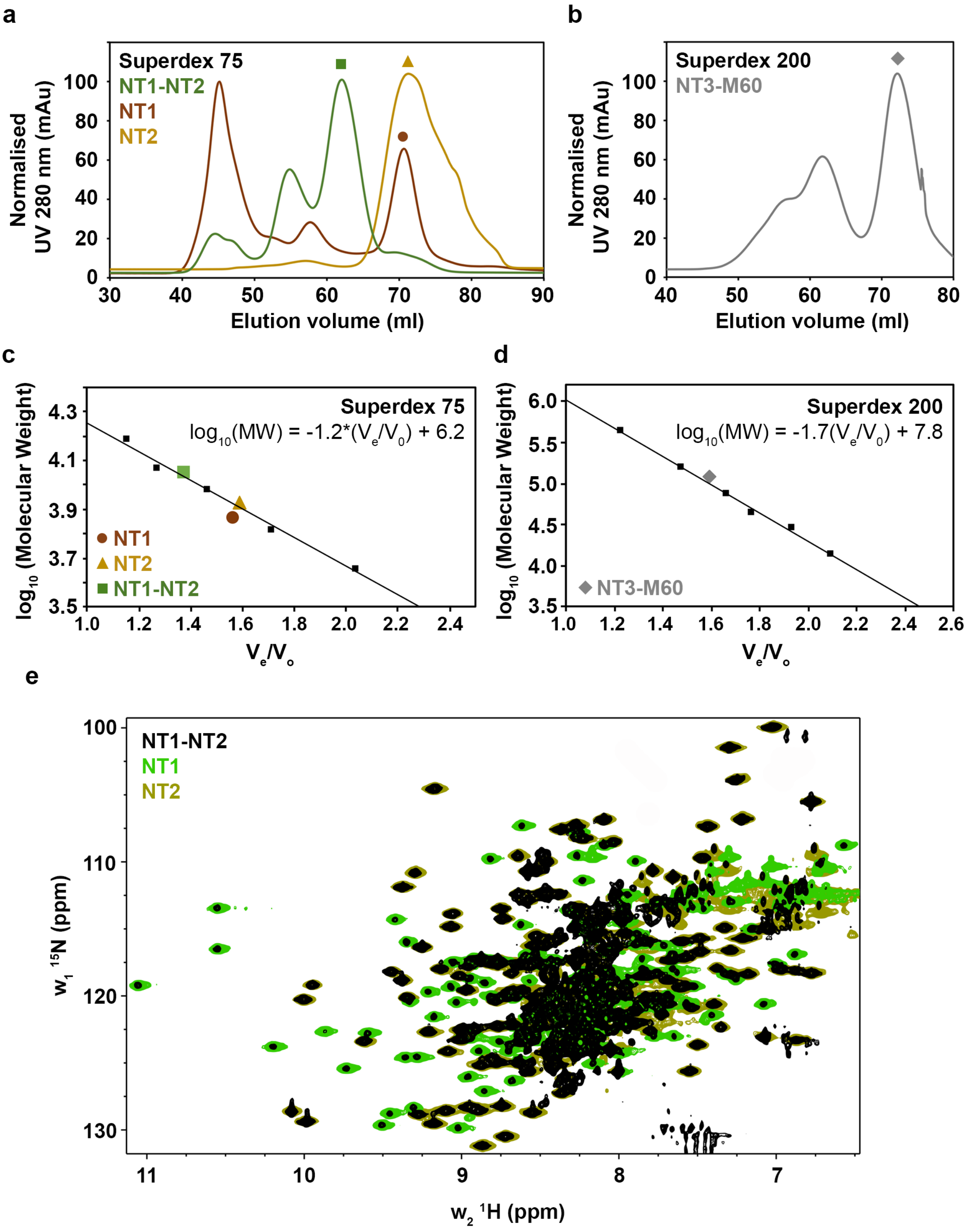

Supplementary Fig. 3 | Folding analysis of SslE subdomain constructs. a-d, Analytical size exclusion chromatography (SEC) of SslE NT1, NT2, NT1-NT2 and NT3-M60 constructs. **a,** Normalised chromatograms of NT1 (brown), NT2 (orange) and NT1-NT2 (green) injected onto a Superdex 75 column. **b,** Normalised chromatogram of NT3-M60 (grey) injected onto a Superdex 200 column. **c,** The V_e/V_o (elution volume/column void volume) for the major peaks of NT1 (brown circle),

NT2 (orange triangle) and NT1-NT2 (green square) were plotted against their Log_{10} molecular weights on a standard curve created using molecular weight standards (GE Healthcare). These profiles show that all constructs are mainly monomeric in solution with calculated molecular weights of 21.8 kDa for NT1 (theoretic mass: 16.9 kDa), 20.7 kDa for NT2 (theoretic mass: 22.8 kDa) and 36.8 kDa for NT1-NT2 (theoretic mass: 40.1 kDa), although other higher molecular weight forms are also observed. **d,** The V_e/V_o (elution volume/column void volume) for the major peak of NT3-M60 (grey diamond) was plotted against its Log_{10} molecular weight on a standard curve created using molecular weight standards (GE Healthcare). This again suggests that the construct is mainly monomeric in solution with a calculated molecular weight of 98.9 kDa (theoretic mass: 121.4 kDa), yet higher molecular weight forms are also observed. **e,** TROSY ^{1}H-^{15}N HSQC spectrum of ^{15}N-labelled SslE NT1-NT2 (black) overlayed with ^{1}H-^{15}N HSQC spectra from the isolated ^{15}N-labelled SslE NT1 (light green) and ^{15}N-labelled NT2 (olive green). All spectra show well dispersed peaks, indicative of folded proteins, with good overlap between peaks from NT1 and NT2 with the NT1-NT2 spectrum.

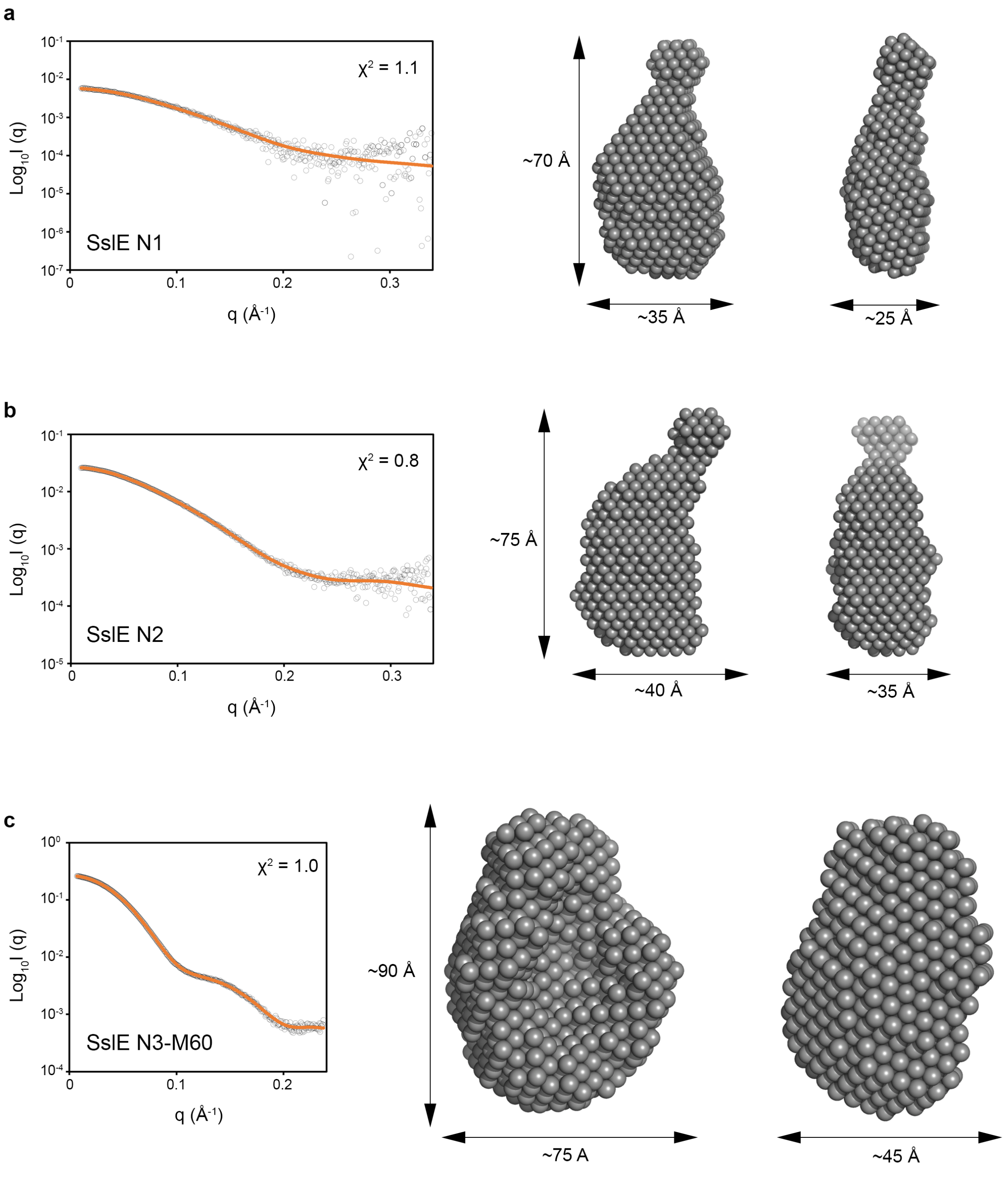

Supplementary Fig. 4 | SAXS analysis of SslE sub-domains. SAXS bead model of SslE **a,** N1, **b,** N2 and **c,** N3-M60 regions at pH 8.0. **c,** Fit of the SAXS bead models (orange line) to the SAXS data (black open circles) is also shown.

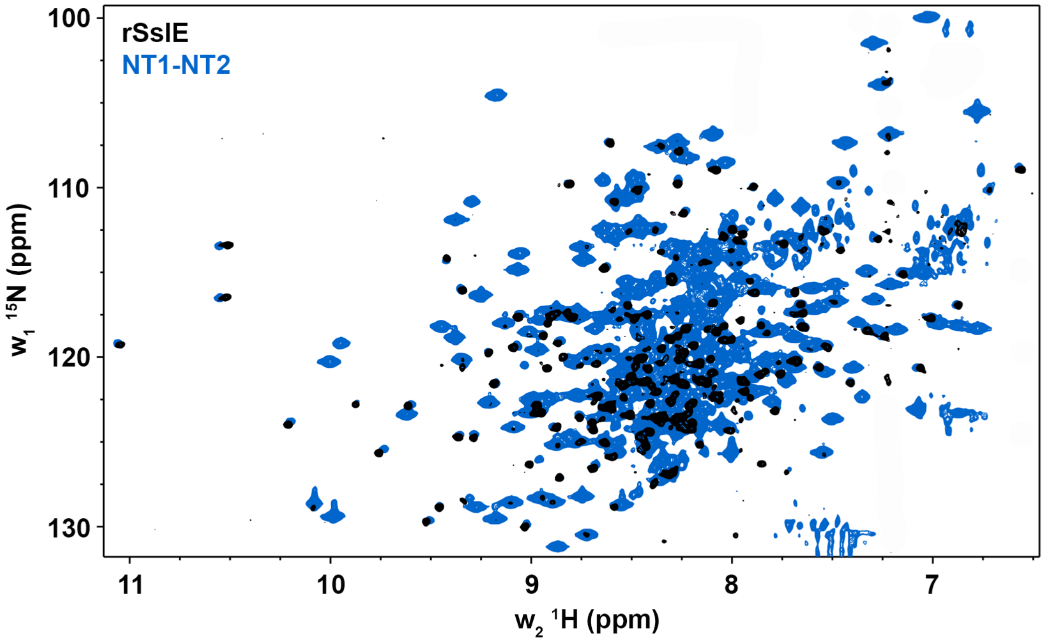

Supplementary Fig. 5 | Comparison of 2D $^{1}H^{15}N$-HSQC spectra of rSslE and SslE NT1-NT2. TROSY ^{1}H-^{15}N HSQC spectrum of ^{15}N-labelled SslE NT1-NT2 (blue) overlayed with a TROSY ^{1}H-^{15}N HSQC spectrum from $^{2}H^{15}N$-labelled rSslE (black).

a

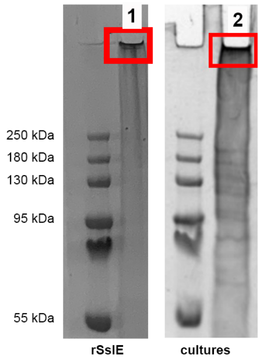

b

```
1      MKTGYLTLGG SLRVTGDITC NDESSDGFTF TPGDKVTCVA GNNTTIATFD TQSEAARSLR AVEKVSFSLE
71     DAQELAGSDN KKSNALSLVT SMNSCPANTE QVCLEFSSVI ESKRFDSLYK QIDLAPEEFK KLVNEEVENN
141    AATDKAPSTH TSPVVPATTP GTKPDLNASF VSANAEQFYQ YQPTEIILSE GRLVDSQGDG VVGVNYYTNS
211    GRGVTGENGE FSFSWGETIS FGIDTFELGS VRGNKSTIAL TELGDEVRGA NIDQLIHRYS KAGQNHTRVV
281    PDEVRKVFAE YPNVINEIIN LSLSNGATLG EGEQVVNLPN EFIEQFKTGQ AKEIDTAICA KTDGCNEARW
351    FSLTTRNVND GQIQGVINKL WGVDTNYKSV SKFHVFHDST NFYGSTGNAR GQAVVNISNA AFPILMARND
421    KNYWLAFGEK RAWDKNELAY ITEAPSIVRP ENVTRETATF NLPFISLGQV GDGKLMVIGN PHYNSILRCP
491    NGYSWNGGVN KDGQCTLNSD PDDMKNFMEN VLRYLSNDRW LPDAKSSMTV GTNLDTVYFK KHGQVLGNSA
561    PFAFHKDFTG ITVKPMTSYG NLNPDEVPLL ILNGFEYVTQ WGSDPYSIPL RADTSKPKLT QQDVTDLIAY
631    MNKGGSVLIM ENVMSNLKEE SASGFVRLLD AAGLSMALNK SVVNNDPQGY PDRVRQRRST PIWVYERYPA
701    VDGKPPYTID DTTKEVIWKY QQENKPDDKP KLEVASWQEE VEGKQVTQFA FIDEADHKTP ESLAAAKQRI
771    LDAFPGLEVC KDSDYHYEVN CLEYRPGTGV PVTGGMYVPQ YTQLDLGADT AKAMLQAADL GTNIQRLYQH
841    ELYFRTNGRQ GERLNSVDLE RLYQNMSVWL WNETKYRYEE GKEDELGFKT FTEFLNCYTN NAYVGTQCSA
911    ELKKSLIDNK MIYGEESSKA GMMNPSYPLN YMEKPLTRLM LGRSWWDLNI KVDVEKYPGA VSEEGQNVTE
981    TISLYSNPTK WFAGNMQSTG LWAPAQKEVT IKSNANVPVT VTVALADDLT GREKHEVALN RPPRVTKTYS
1051   LDASGTVKFK VPYGGLIYIK GDSKDNESAS FTFTGVVKAP FYKDGAWKND LNSPAPLGEL ESDAFVYTAP
1121   KKNLNASNYT GGLKQFANDL DTFASSMNDF YGRNEEDGKH RMFTYKNLTG HKHRFANDVQ ISIGDAHSGY
1191   PVMNSSFSTN STTLPTTPLN DWLIWHEVGH NAAETPLTVP GATEVANNVL ALYMQDRYLG KMNRVADDIT
1261   VAPEYLEESN GQAWARGGAG DRLLMYAQLK EWAEKNFDIK KWYPEGELPK FFSDREGMKG WNLFQLMHRK
1331   ARGDDVGDKT FGGKNYCAES NGNAADTLML CASWVAQTDL SEFFKKWNPG ANAYQLPGAS EMSFEGGVSQ
1401   SAYNTLASLK LPKPEQGPET INKVTEHKMS VEKHHHHHH
```

c

```
1      CDGGGSGSSS DTPPVDSGTG SLPEVKPDPT PNPEPTPEPT PDPEPTPEPT PDPEPTPEPE PEPVPTKTGY
71     LTLGGSLRVT GDITCNDESS DGFTFTPGDK VTCVAGNNTT IATFDTQSEA ARSLRAVEKV SFSLEDAQEL
141    AGSDNKKSNA LSLVTSMNSC PANTEQVCLE FSSVIESKRF DSLYKQIDLA PEEFKKLVNE EVENNAATDK
211    APSTHTSPVV PATTPGTKPD LNASFVSANA EQFYQYQPTE IILSEGRLVD SQGDGVVGVN YYTNSGRGVT
281    GENGEFSFSW GETISFGIDT FELGSVRGNK STIALTELGD EVRGANIDQL IHRYSKAGQN HTRVVPDEVR
351    KVFAEYPNVI NEIINLSLSN GATLGEGEQV VNLPNEFIEQ FKTGQAKEID TAICAKTDGC NEARWFSLTT
421    RNVNDGQIQG VINKLWGVDT NYKSVSKFHV FHDSTNFYGS TGNARGQAVV NISNAAFPIL MARNDKNYWL
491    AFGEKRAWDK NELAYITEAP SIVRPENVTR ETATFNLPFI SLGQVGDGKL MVIGNPHYNS ILRCPNGYSW
561    NGGVNKDGQC TLNSDPDDMK NFMENVLRYL SNDRWLPDAK SSMTVGTNLD TVYFKKHGQV LGNSAPFAFH
631    KDFTGITVKP MTSYGNLNPD EVPLLILNGF EYVTQWGSDP YSIPLRADTS KPKLTQQDVT DLIAYMNKGG
701    SVLIMENVMS NLKEESASGF VRLLDAAGLS MALNKSVVNN DPQGYPDRVR QRRSTPIWVY ERYPAVDGKP
771    PYTIDDTTKE VIWKYQQENK PDDKPKLEVA SWQEEVEGKQ VTQFAFIDEA DHKTPESLAA AKQRILDAFP
841    GLEVCKDSDY HYEVNCLEYR PGTGVPVTGG MYVPQYTQLD LGADTAKAML QAADLGTNIQ RLYQHELYFR
911    TNGRQGERLN SVDLERLYQN MSVWLWNETK YRYEEGKEDE LGFKTFTEFL NCYTNNAYVG TQCSAELKKS
981    LIDNKMIYGE ESSKAGMMNP SYPLNYMEKP LTRLMLGRSW WDLNIKVDVE KYPGAVSEEG QNVTETISLY
1051   SNPTKWFAGN MQSTGLWAPA QKEVTIKSNA NVPVTVTVAL ADDLTGREKH EVALNRPPRV TKTYSLDASG
1121   TVKFKVPYGG LIYIKGDSKD NESASFTFTG VVKAPFYKDG AWKNDLNSPA PLGELESDAF VYTAPKKNLN
1191   ASNYTGGLKQ FANDLDTFAS SMNDFYGRNE EDGKHRMFTY KNLTGHKHRF ANDVQISIGD AHSGYPVMNS
1261   SFSTNSTTLP TTPLNDWLIW HEVGHNAAET PLTVPGATEV ANNVLALYMQ DRYLGKMNRV ADDITVAPEY
1331   LEESNGQAWA RGGAGDRLLM YAQLKEWAEK NFDIKKWYPE GELPKFFSDR EGMKGWNLFQ LMHRKARGDD
1401   VGDKTFGGKN YCAESNGNAA DTLMLCASWV AQTDLSEFFK KWNPGANAYQ LPGASEMSFE GGVSQSAYNT
1471   LASLKLPKPE QGPETINKVT EHKMSVE
```

Supplementary Fig. 6 | Mass Spec analysis of purified SslE aggregates. a, Aggregates formed from recombinant rSslE at pH 4.4 (sample 1) and ring aggregates formed from *E. coli* W strain cultures at pH 5.0 (sample 2) were run on SDS-PAGE. Sample retained in the wells were cut from the gel (red box) and used for mass spec analysis. **b,c,** Summary of peptides identified (yellow) with the sequences for **b**, rSslE and **c**, mature SslE from *E. coli* W strain shown

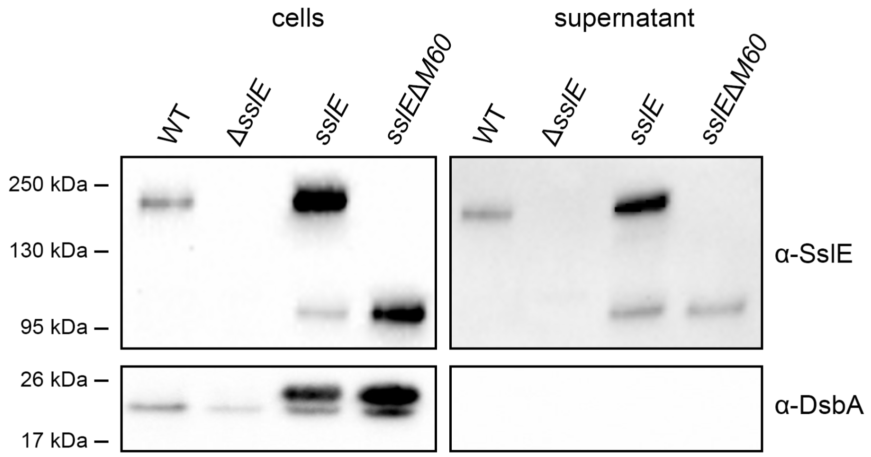

Supplementary Fig. 7 | Secretion of SslE by *E. coli* W and derivatives. Immunoblot of overnight culture of whole cell samples and culture supernatants detected with either anti-rSslE antibody or anti-DsbA antibody, which was used to confirm cell lysis had not occurred in supernatant samples. WT = wild-type; Δ*sslE* = *sslE* mutant; *sslE* = Δ*sslE::sslE*; *sslEΔM60* = Δ*sslE::sslEΔM60*. Each blot was derived from the same experiment and were processed in parallel.

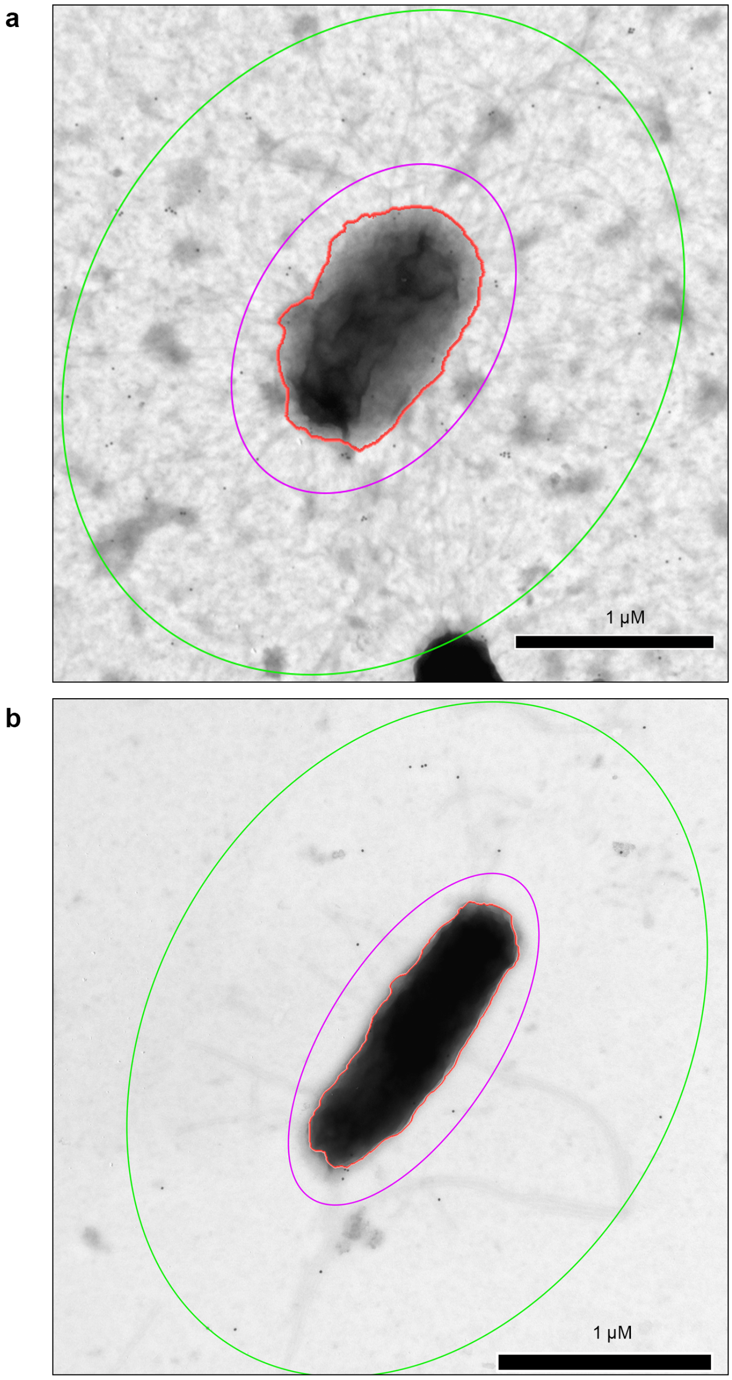

Supplementary Fig. 8 | Immuno-TEM analysis of SslE accumulation in the extracellular milieu. **a,** Wild-type *E. coli* W strain and **b,** Δ*sslE* mutant were washed in citrate-phosphate buffer at pH 5.0 and then reacted with primary α-rSslE antibodies and secondary gold-labelled antibodies on carbon coated nickel grids and then negatively stained with uranyl acetate. Bacteria were masked (red line) and gold particles were counted between ~0.2 µm (purple line) and 1.0 µm (green line) from the bacterium. Scale bars represent 1 µm.

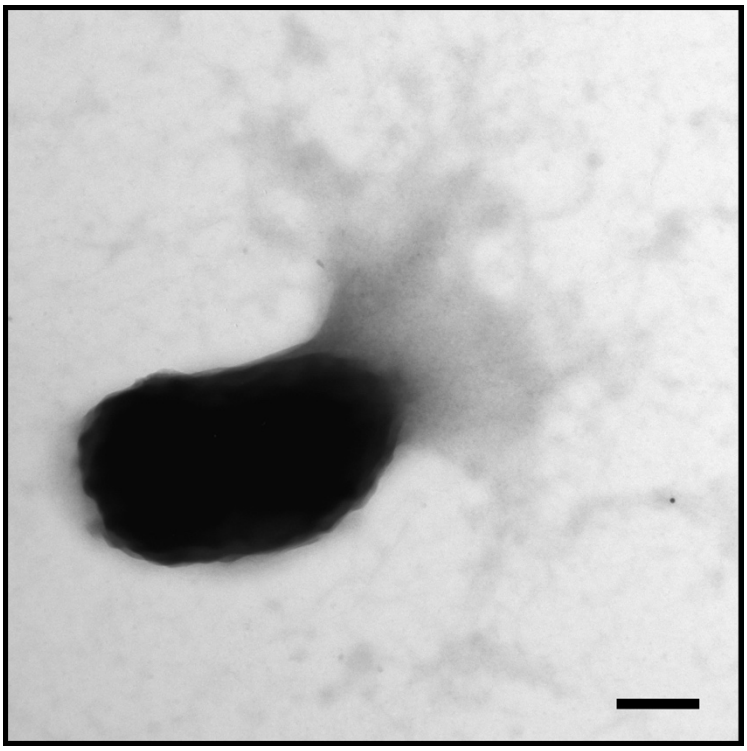

Supplementary Fig. 9 | Immuno-TEM assessment of curli production in wild-type *E. coli* W strain. Bacteria were grown overnight in LB media at pH 5.0 and in addition to analysis with α-rSslE antibodies, the secretion and accumulation of curli fibres was also assessed by immunoelectron microscopy. Bacteria were reacted with polyclonal primary α-CsgA antibodies and secondary gold-labelled antibodies on carbon coated nickel grids and then negatively stained with uranyl acetate. Scale bar represents 200 nm.

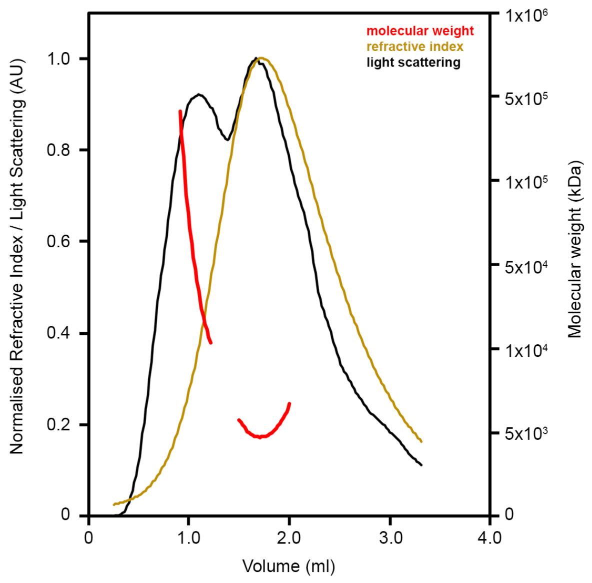

Supplementary Fig. 10 | Mass analysis of rSslE aggregates using RT-MALS. Two species are present, the lager species displaying very high polydispersity. Theoretical mass of rSslE is 160.0 kDa. The signal from the refractive index and light scattering are shown as a yellow and black line, respectively (left, y axis), and the average molecular weight calculated across each peak is shown as a red line (right, y axis).

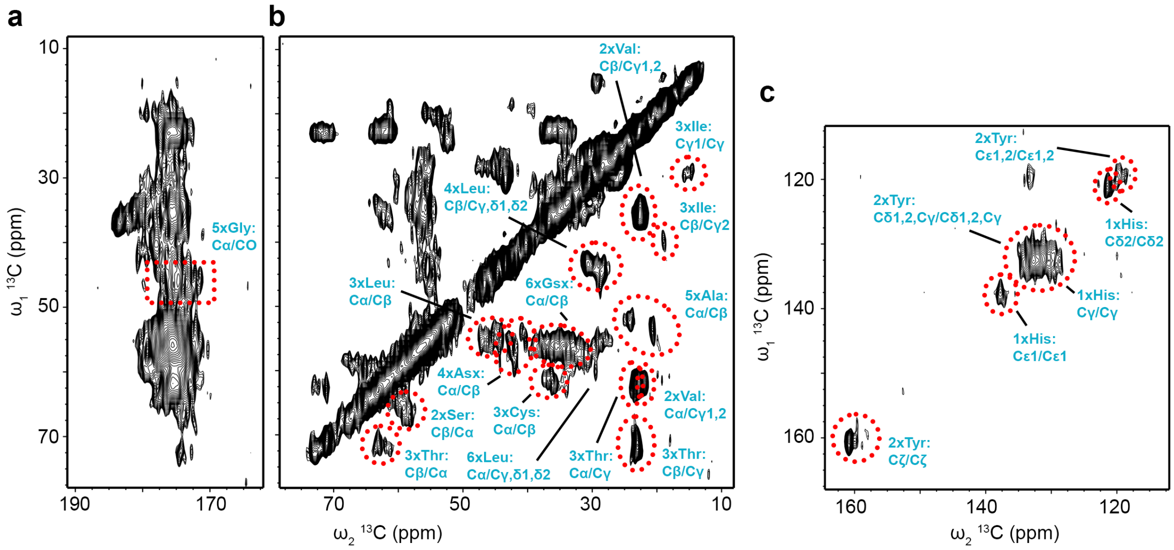

Supplementary Fig. 11 | ssNMR analysis of rSslE aggregates. DARR ssNMR spectrum focussed on the **a,** carbonyl, **b,** aliphatic and **c,** aromatic regions. Likely amino acid type assignments are highlighted with red dashed lines and annotated in teal.

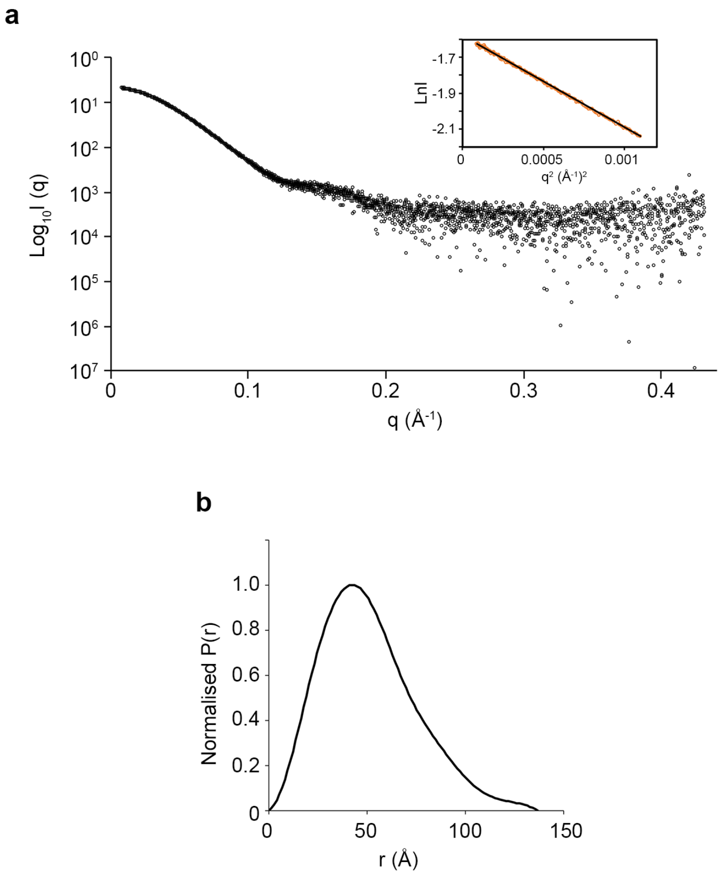

Supplementary Fig. 12 | SEC-SAXS analysis of the monomeric pre-aggregation form of rSslE at pH 4.4. a, Experimental scattering curve of rSslE (black open circles). Inset: Guinier Region (orange open circles) and linear regression (black line) for R_g evaluation. **b,** Shape distribution (*P(r)*) function derived from SAXS analysis for rSslE.

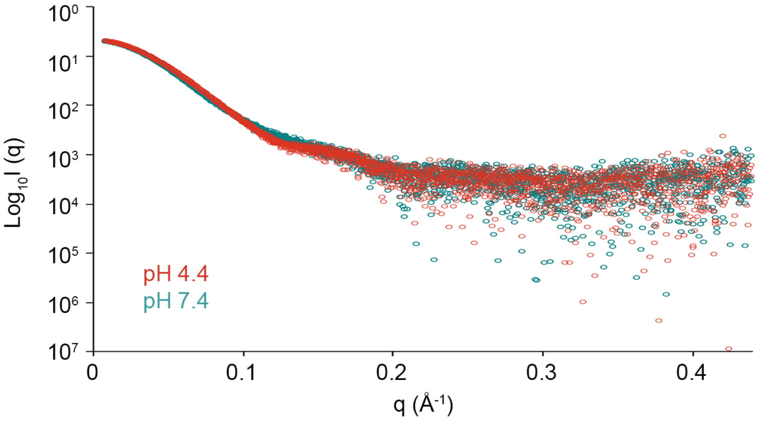

Supplementary Fig. 13 | Comparison of SEC-SAXS scattering curves from monomeric pre-aggregated forms of rSslE at different pH values. SslE at pH 4.4 is red and at 7.4 is teal. Small differences are seen at q-values of approx. 0.13 Å^{-1}.

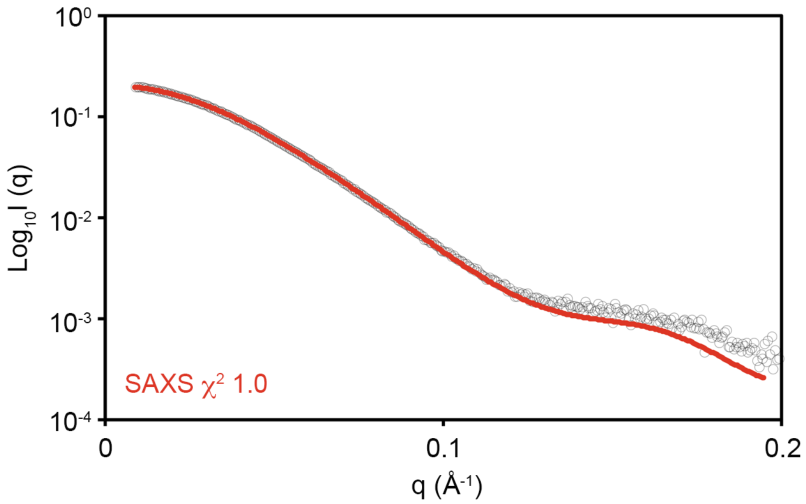

Supplementary Fig. 14 | Fit of the monomeric pre-aggregation rSslE SAXS bead model at pH 4.4 to the corresponding SEC-SAXS data. Bead model is shown as red line and SAXS data as black open circles, with χ^2 of 1.0.

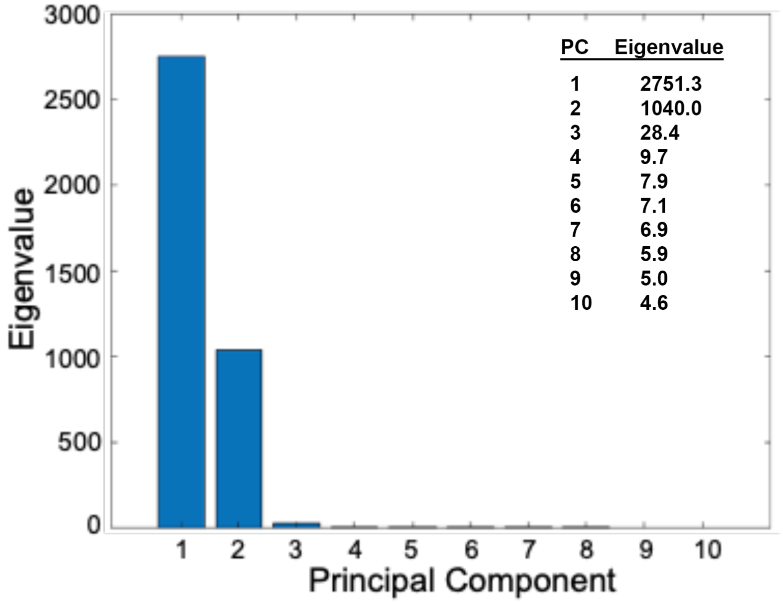

Supplementary Fig. 15 | PCA analysis of rSslE SAXS fibrillation. The Eigenvalues of the first ten principal components obtained after PCA analysis of the fibrillation SAXS dataset.

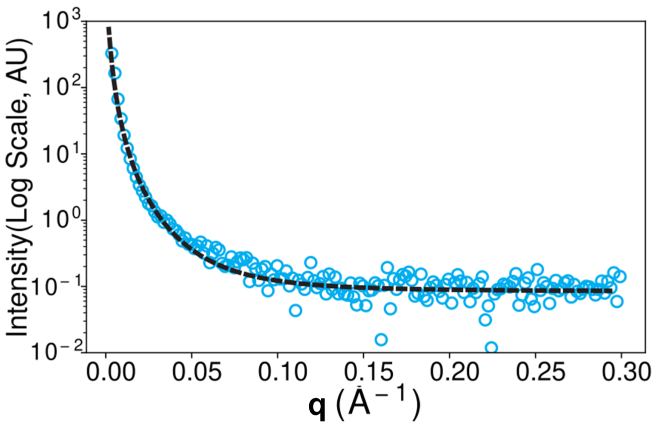

Supplementary Fig. 16 | Fractal fit against rSslE SAXS fibrillation data. The fractal-fit plot (black dashed line) fitted against the decomposed SAXS scattering profile from COSMiCS (blue circles) using a mass-fractal model.

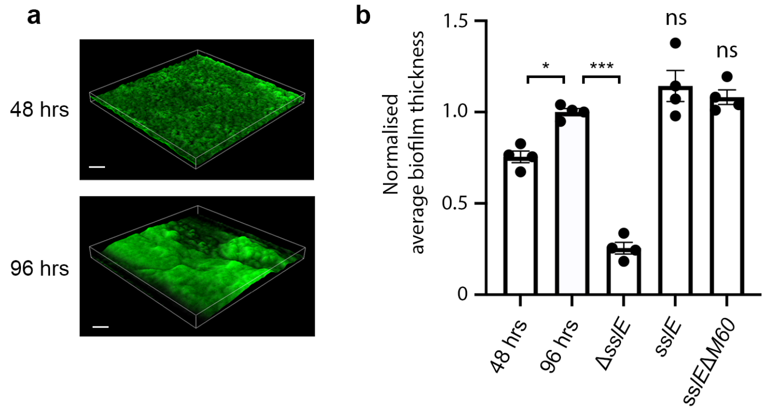

Supplementary Fig. 17 | Analysis of *E. coli* W biofilm growth. a, CLSM images of wild-type *E. coli* W biofilms grown for 48 and 96 hrs and stained with the lipophilic probe FM 1-43 (green). Scale bar represents 20 μm. **b,** Quantification of 3-dimensional biofilm intensity of wild-type *E. coli* W biofilms and its derivatives carried out in ImageJ[1]. $^{*}P$ <0.05; $^{***}P$ <0.001; verses W 96 hrs growth by two-tailed Student's t-test and error bars are the standard error of the mean.

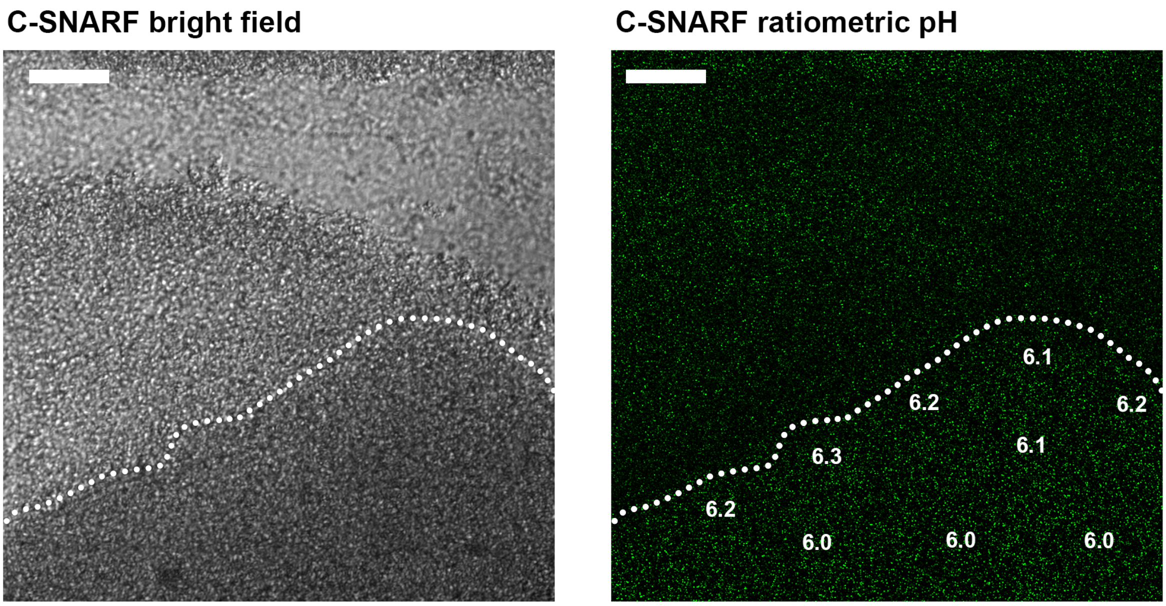

Supplementary Fig. 18 | Analysis of pH across 24 hr old *E. coli* W biofilms. The pH across biofilms grown for 24 hrs was monitored ratiometrically using C-SNARF-4 (green). pH values were calculated over 30 μm^2 boxes and pH values for representative regions are annotated. Dotted line outlines boundary of the biofilm mass. Scale bar represents 20 μm. All data are representative of at least three independent experiments.

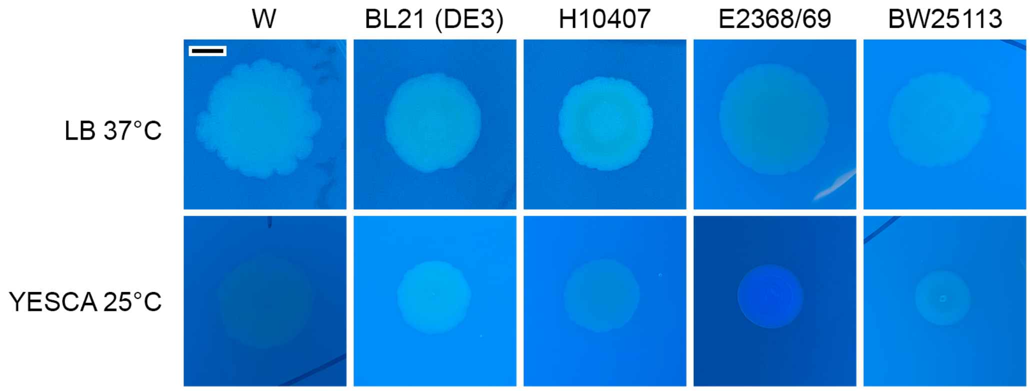

Supplementary Fig. 19 | Cellulose production in *E. coli* strains. Macrocolony biofilm phenotypes of wild-type *E. coli* W, BL21 (DE3), ETEC H10407, EPEC E2368/69 and K12 BW25113 strains after 96 hrs of growth on either LB or YESCA agar containing calcofluor white (Fluorescent Brightener 28) stain. Macrocolonies were visualized under UV light with cellulose production indicated by colony fluorescence. Scale bar represents 5 mm. Previous calcofluor white analysis of *E. coli* W and ETEC H10407 macrocolonies[2] had been incorrectly analyzed due to the calcofluor white reagent containing Evans Blue dye, which had unintentionally stained protein aggregates.

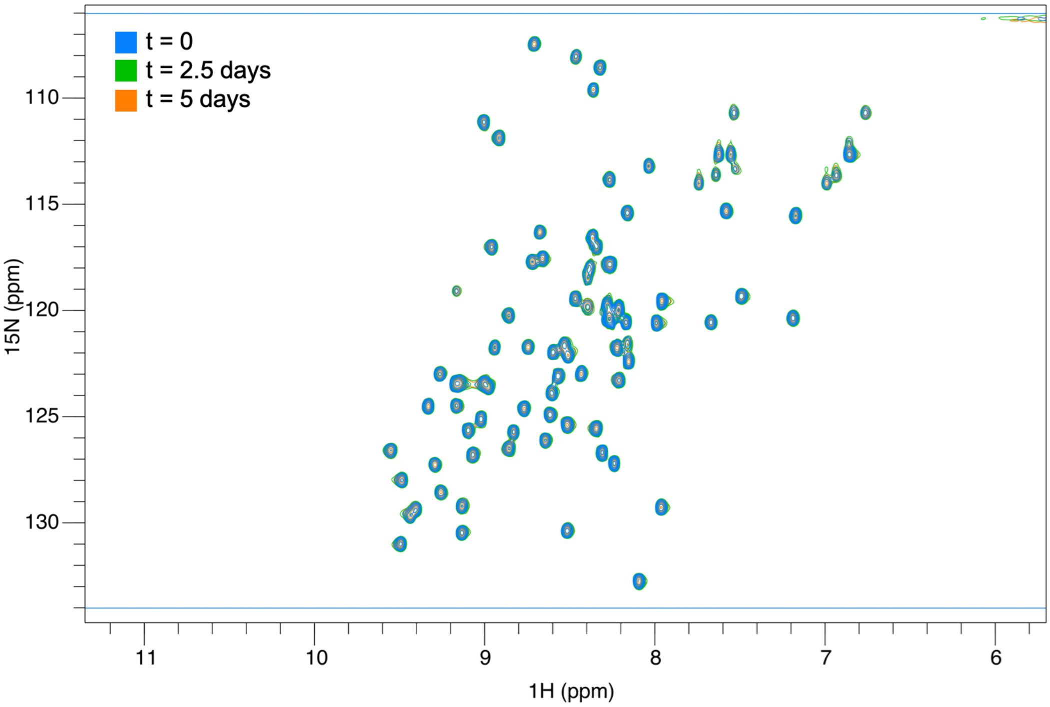

Supplementary Fig. 20 | 2D $^{1}H^{15}N$-HSQC analysis of RgpB-CTD stability at pH 6.0. Spectra were recorded after 0, 2.5 and 5 days incubation at 37°C. Excellent dispersion and narrow line widths can be observed for the majority of peaks suggesting this is a well folded domain. No new peaks are observed following 5 days incubation showing that the protein is stable in these conditions.

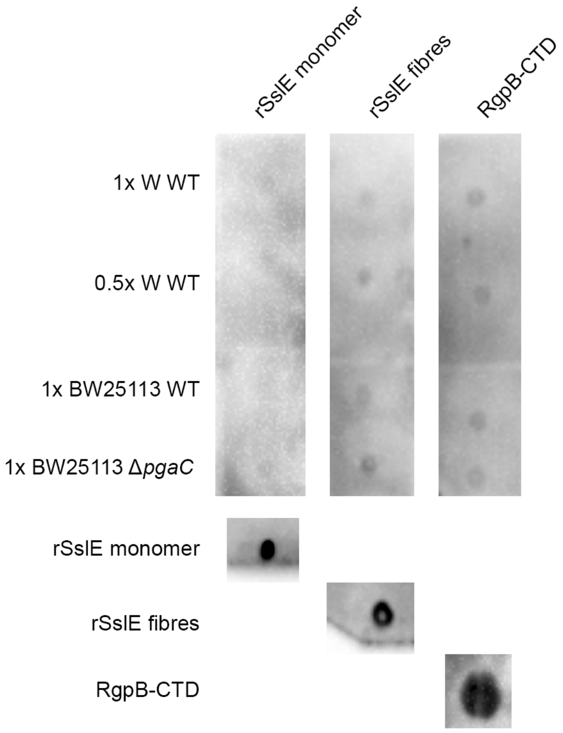

Supplementary Fig. 21 | Dot-blot analysis of bacterial PNAG interactions with rSslE. Dot-blots of partially purified PNAG from wild-type *E. coli* W, wild-type *E. coli* BW25113 and *E. coli* BW25113 Δ*pgaC* mutant strains overlayed with His_6-tagged rSslE monomers, His_6-tagged rSslE fibres and His_6-tagged *P. gingivalis* RgpB-CTD negative control, at pH 6.0 and detected with anti-His_6 antibody. Spotted monomeric rSslE, rSslE fibres and RgpB-CTD were also used as positive controls. Each blot (rSslE monomer, rSslE fibres, RgpB-CTD) was derived from the same experiment and were processed in parallel.

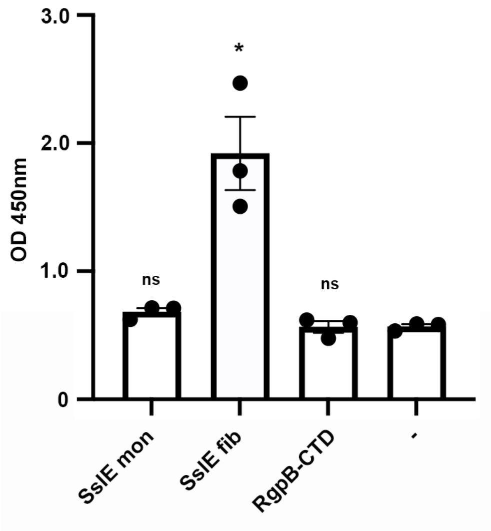

Supplementary Fig. 22 | Binding properties of rSslE and cellulose. ELISA analysis of binding between cellulose discs produced by *K. rhaeticus* and His_6-tagged rSslE monomers, His_6-tagged rSslE fibres and His_6-tagged *P. gingivalis* RgpB-CTD control, at pH 6.0 and detected with anti-His_6 antibody. BSA-coated wells were used as controls. $^{*}P$ <0.05; verses control empty well (-) by two-tailed Student's test. Error bars are the standard error of the mean. All data are representative of at least three independent experiments performed in triplicate.

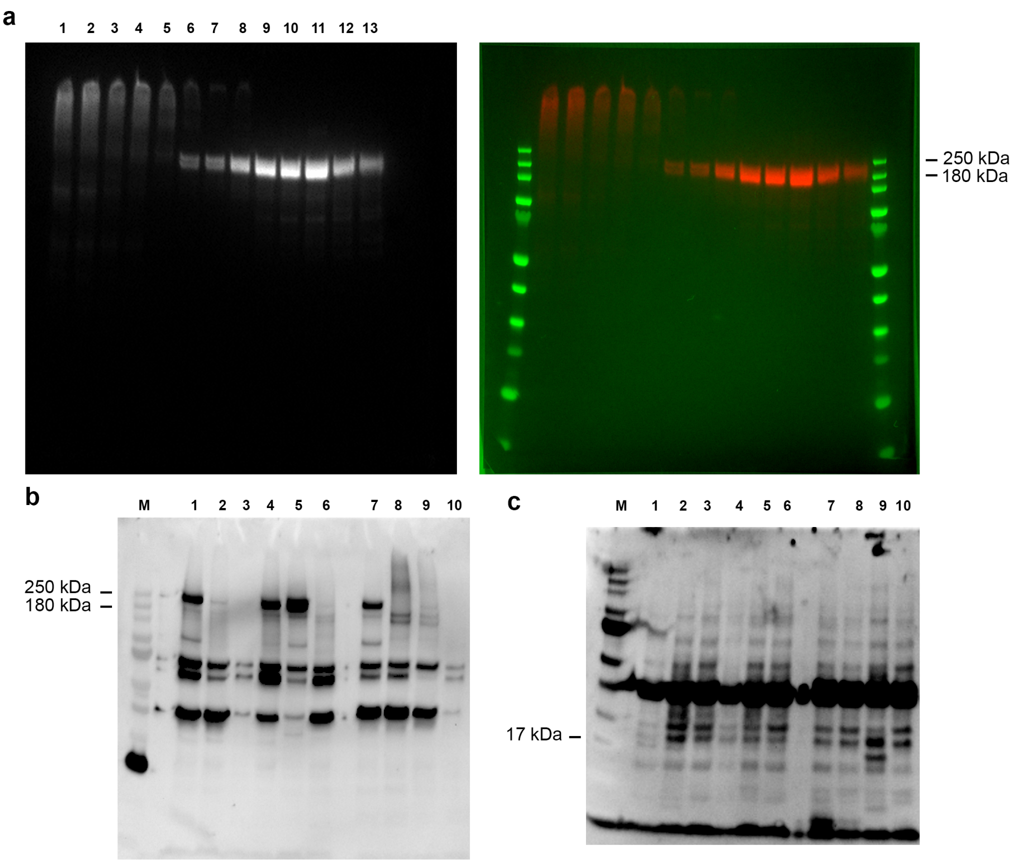

Supplementary Fig. 23 | Raw immunoblot images. a, Uncropped and unprocessed immunoblot shown in Fig. 2c: rSslE incubated in citrate-phosphate buffer from pH 3.8 to 4.8 and detected with anti-His_6 antibody. Right hand image shows raw immuoblot, left image is overlaid with molecular weight marker. Lanes 1-13 correspond to pH values 3.0, 3.2, 3.4, 3.6, 3.8, 4.0, 4.2, 4.4, 4.6, 4.8, 5.0, 6.0 and 7.0. **b,c,** Uncropped and unprocessed immunoblots shown in Fig. 6d: immunoblot analysis of whole cells detected with **b,** anti-rSslE antibody or **c,** anti-CsgA antibody. Lanes M, molecular weight markers; 1, *E. coli* W WT 37°C; 2, *E. coli* W WT 25°C; 3, *E. coli* W Δ*sslE* 37°C; 4, *E. coli* BL21 (DE3) WT 37°C; 5, *E. coli* H10407 WT 37°C; 6: *E. coli* H10407 Δ*sslE* 37°C; 7, *E. coli* EPEC WT 37°C; 8: *E. coli* EPEC Δ*sslE* 37°C; 9, *E. coli* EPEC WT 25°C; 10, *E. coli* EPEC Δ*csgA* 25°C.

Supplementary Tables

Supplementary Table 1: SslE SAXS data and refinement statistics

	SslE N1	SslE N2	SslE N3-M60	rSslE solution data		COSMiCS rSslE fibrillation data	
**SAXS data collection**				pH 7.4	pH 4.4	monomer	fibre
Beamline	DLS B21	DLS B21	DLS B21	DLS B21	DLS B21	DLS B21	DLS B21
Wavelength (Å)	1.0	1.0	1.0	1.0	1.0	1.0	1.0
q Range ($Å^{-1}$)	0.003 to 0.44	0.003 to 0.44	0.003 to 0.44	0.003 to 0.44	0.003 to 0.44	0.003 to 0.44	0.003 to 0.44
**Structural parameters**							
I(0)	0.000575 ± 1.7e-05	0.0266 ± 2.0e-05	0.000575 ± 1.7e-05	0.203 ± 1.5e-04	0.206 ± 1.6e-04	-	-
R_g (nm) (from Guinier)	2.01 ± 0.05	2.20 ± 0.04	3.37 ± 0.01	4.03 ± 0.05	3.92 ± 0.05	3.92	-
R_g (nm) (from P(r))	2.06 ± 0.01	2.26 ± 0.00	3.39 ± 0.00	4.07 ± 0.01	3.97 ± 0.01	3.98	51.1
D_{max} (nm) (from P(r))	7.0	7.7	10.5	14.1	13.7	13.3	140
DAMMIN model χ^2 fit	1.13	0.84	1.04	1.19	0.96	-	-
DAMMIN models NSD (20 models)	0.49	0.42	0.63	0.56	0.45	-	-
EM model χ^2 fit	-	-	-	113.70	-	-	-
**Molecular mass determination**							
MW (kDa) (from sequence)	16886	22792	121391	159986	159986	159986	-
MW (kDa) (from SAXS)	17096	23283	122275	151106	158202	146800 (Bayesian Estimate)	-

Supplementary Table 2: circular dichroism data processing.

**Secondary structure type**	**% secondary structure**		**Difference**
	pH 4.4	pH 7.4	
α-helix	21.7	29.9	8.2
β-sheet	17.3	12.2	5.1
β-turns	14.2	13.1	1.1
Random coil	46.8	44.7	2.1
NRMSD	0.013	0.007	-

Supplementary Table 3: Primers, plasmids and strains used in this study

Primer	Description	Sequence (5' to 3')
BD1	Recombinant expression of *P. gingivalis* W50 RgpB-CTD F	GACGACGACAAGATGGGTACATCTATTGCCGACGTAG
BD2	Recombinant expression of *P. gingivalis* W50 RpgB-CTD R	GAGGAGAAGCCCGGTTACTTCACTATAACCTTTTCTG TATACGTC
PC3	Gene KO - *E. coli* JW5925-1 *sslE* downstream	TTATTTCATGCCGGATGCGGCGTGAACGCCTTATCCG GCATACAGGATTATGTAGGC
PC4	Gene KO -*E. coli* JW5925-1 *sslE* upstream	TTTCTCCCAGTTACGAATTTTTAACATTTGTCAGTT GCGTTATTAATTCCGGGGA
PC5	*sslE* complementing plasmid F	CCGGGCTAGCTATCAATGATGTCGTTTTCTTAAGAAT GGA
PC6	Full *sslE* complementing plasmid R	CCGGAAGCTTTTACTCGACAGACATCTTATGCTCGGT AACCT
PC7	*sslEΔM60* complementing plasmid R	CCGGAAGCTTTTACGGGTTCATCATGCCCGCTTTGCTG

Plasmid	Description	Reference
pPC1	pOPINE expression of *E. coli* W SslE (full) residues 67-1497	[3]
pPC2	pET28b expression of *E. coli* SslE (NT1) residues 67-211	This study
pPC3	pET28b expression of *E. coli* SslE (NT2) residues 230-425	This study
pPC4	pET28b expression of *E. coli* SslE (NT1-NT2) residues 67-425	This study
pPC5	pET28b expression of *E. coli* SslE (NT3-M60) residues 426-1497	This study
pBD1	pET46 Ek/LIC expression of *P. gingivalis* W50 RpgB (CTD) residues 662-736	This study
pOPINE	Expression vector	[4]
pET28b	Expression vector	Novagen
pET46 Ek/LIC	Expression vector	Novagen
pKD46	Red recombinase expression	[5]
pBAD18-Cm	Complementation plasmid	[6]
pCPC1	Full *sslE* complementing plasmid	This study
pCPC2	*sslEΔM60* complementing plasmid	This study

E. coli strain	Serogroup/ genotype	Reference
DH5a	*F- Φ80lacZΔM15 Δ(lacZYA-argF) U169 recA1 endA1 hsdR17(rk-, mk+) phoA supE44 thi-1 gyrA96 relA1 λ-*	Invitrogen
BL21 (DE3)	*fhuA2 [lon] ompT gal (λ DE3) [dcm] ΔhsdS*<br>*λ DE3 = λ sBamHIo ΔEcoRI-B int::(lacI::PlacUV5::T7 gene1) i21 Δnin5*	
Shuffle T7	*fhuA2 lacZ::T7 gene1* [lon] *ompT ahpC gal λatt::*pNEB3-r1-*cDsbC* (Spec^R, *lacI*^{q}) Δ*trxB sulA11 R(mcr-73::miniTn10*--Tet^S)2 [dcm] *R(zgb-210::Tn10* --Tet^S) *endA1 Δgor* Δ*(mcrC-mrr)114::IS10*	NEB
BW25113	F-, *Δ(araD-araB)567, ΔlacZ4787*(::rrnB-3), *λ*$^-$, *rph-1*, *Δ(rhaD-rhaB)568*, *hsdR514*	[7]
JW5925-1	*sslE* gene disrupted with a kan^R cassette in BW25113	[7]
JW1007-1	*pgaC* gene disrupted with a kan^R cassette in BW25113	[7]
JW2039-1	*wcaF* gene disrupted with a kan^R cassette in BW25113	[7]
W	Serotype (O6:K-:H49) CA$^+$	[8]

(ATCC 35401)		
W Δ*sslE*	*sslE* gene disrupted with a kan^R cassette	This study
H10407	Serotype O78:H11	[9]
(ATCC 35401)		
H10407 Δ*sslE*	*sslE* gene disrupted with a kan^R cassette	This study
E2348/69	Serotype O127:H6	[10, 11]
E2348/69 Δ*csgA*	*csgA* gene disrupted with a kan^R cassette	[11]
E2348/69 Δ*csgA*/Δ*bcsA*	*csgA* gene disrupted with a kan^R cassette; *bcsA* gene disrupted with a chloro^R cassette	[11]
E2348/69 Δ*sslE*	*sslE* gene deletion	[12]

Supplementary Table 4: Synthetic genes

Gene	Description	Sequence (5' to 3')
gPC2	*E. coli* SslE (NT1) residues 67-211	CCATGGTCAAAACCGGTTATCTGACCTTAGGTGGTAGCCTGCGTGTTACCGGTGATATT<br>ACCTGTAATGATGAAAGCTCAGATGGCTTTACCTTTACACCGGGTGATAAAGTTACCTG<br>TGTTGCAGGTAATAATACCACCATTGCAACCTTTGATACCCAGAGCGAAGCAGCACGTA<br>GTCTGCGTGCAGTTGAAAAAGTTAGCTTTAGCCTGGAAGATGCACAAGAACTGGCAGGT<br>AGCGATAACAAAAAAAGCAATGCACTGAGCCTGGTTACCAGCATGAATAGCTGTCCGGC<br>AAATACCGAACAGGTTTGTCTGGAATTTAGCAGCGTTATTGAAAGCAAACGTTTCGATA<br>GCCTGTATAAGCAGATTGATCTGGCACCGGAAGAATTCAAAAAACTGGTTAATGAAGAA<br>GTGGAAAACAACGCAGCAACCGATAAAGCACTCGAG
gPC3	*E. coli* SslE (NT2) residues 230-425	CCATGGTCGATCTGAATGCAAGCTTTGTTAGCGCAAATGCCGAACAGTTTTATCAGTAT<br>CAGCCGACCGAAATTATTCTGAGCGAAGGTCGTCTGGTTGATAGCCAAGGTGATGGTGT<br>TGTTGGTGTTAACTATTACACCAATAGCGGTCGTGGTGTTACCGGTGAAAATGGTGAAT<br>TTAGCTTTAGCTGGGGTGAAACCATTAGCTTTGGTATTGATACCTTTGAACTGGGTAGC<br>GTGCGTGGTAATAAAAGCACCATTGCACTGACCGAACTGGGTGATGAAGTTCGTGGTGC<br>AAATATTGATCAACTGATCCACCGTTATAGCAAAGCAGGTCAGAATCATACCCGTGTTG<br>TTCCGGATGAAGTGCGTAAAGTTTTTGCAGAATATCCGAACGTGATCAACGAAATTATC<br>AATCTGAGCCTGTCAAATGGTGCAACCTTAGGTGAAGGTGAACAGGTTGTTAATCTGCC<br>GAACGAATTCATCGAACAGTTCAAAACCGGTCAGGCCAAAGAAATTGATACCGCAATTT<br>GTGCAAAAACCGATGGTTGTAATGAAGCACGTTGGTTTAGCCTGACCACACGTAATGTT<br>AATGATCTCGAG
gPC4	*E. coli* SslE (NT1-NT2) residues 67-425	CCATGGTCAAAACCGGTTATCTGACCTTAGGTGGTAGCCTGCGTGTTACCGGTGATATT<br>ACCTGTAATGATGAAAGCTCAGATGGCTTTACCTTTACACCGGGTGATAAAGTTACCTG<br>TGTTGCAGGTAATAATACCACCATTGCAACCTTTGATACCCAGAGCGAAGCAGCACGTA<br>GTCTGCGTGCAGTTGAAAAAGTTAGCTTTAGCCTGGAAGATGCACAAGAACTGGCAGGT<br>AGCGATAACAAAAAAAGCAATGCACTGAGCCTGGTTACCAGCATGAATAGCTGTCCGGC<br>AAATACCGAACAGGTTTGTCTGGAATTTAGCAGCGTTATTGAAAGCAAACGTTTCGATA<br>GCCTGTATAAGCAGATTGATCTGGCACCGGAAGAATTCAAAAAACTGGTTAATGAAGAA<br>GTGGAAAACAACGCAGCAACCGATAAAGCACCGAGCACACATACCAGTCCGGTTGTTCC<br>GGCAACCACACCGGGTACAAAACCGGATCTGAATGCAAGCTTTGTTAGCGCAAATGCGG<br>AACAGTTTTATCAGTATCAGCCGACCGAAATTATTCTGAGCGAAGGTCGTCTGGTTGAT<br>AGCCAAGGTGATGGTGTTGTTGGTGTTAACTATTACACCAATAGCGGTCGTGGTGTGAC<br>CGGTGAAAATGGTGAATTTAGCTTTTCATGGGGTGAAACCATTAGCTTTGGCATCGATA<br>CCTTTGAACTGGGTAGCGTTCGTGGTAATAAAAGTACCATTGCACTGACCGAACTGGGT<br>GATGAAGTTCGTGGTGCAAATATTGATCAACTGATCCACCGTTATAGCAAAGCAGGTCA<br>GAATCATACCCGTGTTGTGCCGGATGAAGTGCGTAAAGTTTTTGCAGAATATCCGAACG<br>TGATCAACGAAATTATCAATCTGAGCCTGAGCAATGGTGCAACCTTAGGCGAAGGTGAA<br>CAGGTTGTTAATCTGCCGAATGAATTCATCGAACAGTTTAAAACCGGTCAGGCCAAAGA<br>AATTGATACCGCAATTTGTGCAAAAACCGATGGTTGTAATGAAGCACGTTGGTTTAGCC<br>TGACCACACGTAATGTTAATGATCTCGAG

gPC5	*E. coli* SslE<br>(NT3-M60)<br>residues 426-1497	CCATGGTCGGTCAGATTCAGGGTGTTATTAACAAACTGTGGGGTGTCGATACCAACTAT<br>AAAAGCGTTAGCAAATTTCACGTGTTCCACGATAGCACCAACTTTTATGGTAGCACCGG<br>TAATGCACGTGGTCAGGCAGTTGTGAATATTAGCAATGCAGCATTTCCGATTCTGATGG<br>CACGTAACGATAAAAACTATTGGCTGGCCTTTGGTGAAAAACGTGCATGGGATAAAAAT<br>GAGCTGGCCTATATTACCGAAGCACCGAGCATTGTTCGTCCGGAAAATGTTACCCGTGA<br>AACCGCAACCTTTAATCTGCCGTTTATTAGCCTGGGTCAAGTTGGTGATGGTAAACTGA<br>TGGTTATTGGTAACCCGCATTATAACAGCATTCTGCGTTGTCCGAATGGTTATAGCTGG<br>AATGGTGGTGTTAATAAAGATGGTCAGTGTACCCTGAATAGCGATCCGGATGATATGAA<br>AAACTTCATGGAAAATGTGCTGCGCTATCTGAGCAATGATCGTTGGCTGCCGGATGCAA<br>AAAGCAGCATGACCGTTGGCACCAATCTGGATACCGTTTATTTCAAAAAACATGGTCAG<br>GTGCTGGGTAATAGCGCACCGTTTGCATTTCATAAAGATTTTACCGGCATTACCGTTAA<br>ACCGATGACCAGCTATGGTAATCTGAACCCGGATGAAGTTCCGCTGCTGATTCTGAATG<br>GTTTTGAATATGTTACCCAGTGGGGTAGTGATCCGTATAGCATTCCGCTGCGTGCAGAT<br>ACCAGCAAACCGAAACTGACCCAGCAGGATGTTACCGATCTGATTGCATATATGAATAA<br>AGGTGGTAGCGTGCTGATTATGGAAAACGTTATGAGCAACCTGAAAGAAGAAAGCGCAA<br>GCGGTTTTGTTCGTCTGCTGGATGCAGCAGGTCTGAGCATGGCACTGAATAAAAGTGTG<br>GTTAATAATGATCCGCAGGGTTATCCTGATCGTGTTCGTCAGCGTCGTAGCACCCCGAT<br>TTGGGTTTATGAACGTTATCCGGCAGTTGATGGCAAACCGCCTTATACCATTGATGATA<br>CCACCAAAGAAGTGATCTGGAAGTATCAGCAAGAAAACAAACCTGATGATAAGCCGAAA<br>CTGGAAGTTGCAAGCTGGCAAGAAGAAGTTGAAGGTAAACAGGTGACCCAGTTTGCCTT<br>TATTGATGAAGCAGATCATAAAACACCGGAAAGCCTGGCAGCAGCAAAACAGCGTATTC<br>TGGATGCATTTCCTGGTCTGGAAGTTTGTAAAGATAGCGATTATCACTATGAGGTGAAC<br>TGCCTGGAATATCGTCCTGGCACCGGTGTTCCGGTTACCGGTGGTATGTATGTTCCGCA<br>GTATACCCAGCTGGATCTGGGTGCCGATACCGCAAAAGCAATGCTGCAGGCAGCAGATC<br>TGGGCACCAATATTCAGCGTCTGTATCAGCATGAACTGTATTTTCGTACCAATGGTCGT<br>CAGGGTGAACGTCTGAATAGTGTGGATCTGGAACGCCTGTATCAGAATATGAGCGTTTG<br>GCTGTGGAACGAAACCAAATATCGTTATGAAGAGGGCAAAGAAGATGAGCTGGGCTTTA<br>AAACCTTTACCGAATTTCTGAACTGCTACACCAATAATGCATATGTTGGCACCCAGTGT<br>AGCGCAGAACTGAAAAAAAGTCTGATCGACAACAAAATGATCTACGGTGAGGAAAGCAG<br>CAAAGCCGGTATGATGAATCCGAGCTATCCGCTGAATTATATGGAAAAACCGCTGACAC<br>GTCTGATGCTGGGTCGTAGTTGGTGGGATCTGAACATTAAAGTTGACGTGGAAAAATAT<br>CCGGGTGCAGTTAGCGAAGAAGGTCAGAACGTTACCGAAACCATTAGCCTGTATAGCAA<br>TCCGACCAAATGGTTTGCAGGTAATATGCAGAGCACCGGTCTGTGGGCACCAGCACAGA<br>AAGAAGTTACCATTAAAAGTAATGCCAATGTGCCGGTGACCGTTACCGTTGCACTGGCA<br>GATGATCTGACCGGTCGTGAAAAACATGAAGTTGCACTGAATCGTCCGCCTCGTGTTAC<br>CAAAACCTATTCATTAGATGCAAGCGGCACCGTGAAATTCAAAGTTCCGTATGGTGGTC<br>TGATCTATATCAAAGGTGATAGCAAAGATAATGAGAGCGCCAGCTTTACCTTTACAGGT<br>GTTGTTAAAGCACCGTTCTATAAAGACGGTGCCTGGAAAAACGATCTGAATTCTCCGGC<br>ACCGCTGGGTGAACTGGAAAGTGATGCATTTGTTTATACCGCACCGAAAAAGAATCTGA<br>ACGCAAGCAATTATACCGGTGGCCTGAAACAGTTTGCAAATGATCTGGACACCTTTGCC<br>AGCTCCATGAATGATTTTTATGGTCGCAATGAAGAGGACGGCAAACATCGTATGTTTAC<br>CTATAAAAACCTGACGGGTCATAAACACCGTTTTGCCAATGATGTGCAGATTAGCATTG<br>GTGATGCACATAGCGGTTATCCGGTTATGAATAGCAGCTTTAGCACCAATAGCACCACA<br>CTGCCGACCACACCGCTGAACGATTGGCTGATTTGGCATGAAGTGGGTCATAATGCAGC<br>AGAAACTCCGCTGACCGTTCCGGGTGCCACCGAAGTTGCCAATAATGTTCTGGCACTGT<br>ATATGCAGGATCGTTATCTGGGTAAAATGAATCGTGTTGCCGATGATATTACCGTGGCA<br>CCGGAATATCTGGAAGAAAGCAATGGTCAGGCCTGGGCACGTGGTGGTGCGGGTGATCG<br>CCTGCTGATGTATGCACAGCTGAAAGAATGGGCAGAAAAAAACTTCGACATCAAAAAGT<br>GGTATCCGGAAGGCGAACTGCCGAAATTTTTCAGCGATCGTGAAGGTATGAAAGGCTGG<br>AACCTGTTTCAACTGATGCATCGTAAAGCCCGTGGTGATGATGTTGGTGATAAAACATT<br>TGGTGGCAAAAACTACTGTGCCGAAAGTAATGGTAATGCAGCTGATACCCTGATGCTGT<br>GTGCGAGCTGGGTTGCACAGACCGATCTGTCAGAATTCTTCAAAAAATGGAATCCTGGT<br>GCCAATGCATATCAGTTACCGGGTGCAAGCGAAATGAGCTTTGAAGGTGGTGTGAGCCA<br>GAGCGCATATAATACCCTGGCAAGCCTGAAACTGCCTAAACCTGAACAGGGTCCTGAAA<br>CAATCAACAAAGTTACCGAACATAAGATGAGCGTGGAACTCGAG

*Restriction sites are underlined

Supplementary References

1. Schindelin J*, et al.* Fiji: an open-source platform for biological-image analysis. *Nat Methods* **9**, 676-682 (2012).

2. Corsini PM*, et al.* Molecular and cellular insight into Escherichia coli SslE and its role during biofilm maturation. *bioRxiv*, 2021.2002.2007.430137 (2021).

3. Rehman S*, et al.* Structure and functional analysis of the Legionella pneumophila chitinase ChiA reveals a novel mechanism of metal-dependent mucin degradation. *PLoS Pathog* **16**, e1008342 (2020).

4. Berrow NS*, et al.* A versatile ligation-independent cloning method suitable for high-throughput expression screening applications. *Nucleic Acids Res* **35**, e45 (2007).

5. Datsenko KA, Wanner BL. One-step inactivation of chromosomal genes in Escherichia coli K-12 using PCR products. *Proc Natl Acad Sci U S A* **97**, 6640-6645 (2000).

6. Guzman LM, Belin D, Carson MJ, Beckwith J. Tight regulation, modulation, and high-level expression by vectors containing the arabinose PBAD promoter. *J Bacteriol* **177**, 4121-4130 (1995).

7. Baba T*, et al.* Construction of Escherichia coli K-12 in-frame, single-gene knockout mutants: the Keio collection. *Mol Syst Biol* **2**, 2006 0008 (2006).

8. Uri J, Valu G, Bekesi I. Production of 6-Aminopenicillanic Acid by Dermatophytes. *Nature* **200**, 896-897 (1963).

9. Skerman FJ, Formal SB, Falkow S. Plasmid-associated enterotoxin production in a strain of Escherichia coli isolated from humans. *Infect Immun* **5**, 622-624 (1972).

10. Levine MM*, et al.* Escherichia coli strains that cause diarrhoea but do not produce heat-labile or heat-stable enterotoxins and are non-invasive. *Lancet* **1**, 1119-1122 (1978).

11. Saldana Z*, et al.* Synergistic role of curli and cellulose in cell adherence and biofilm formation of attaching and effacing Escherichia coli and identification of Fis as a negative regulator of curli. *Environ Microbiol* **11**, 992-1006 (2009).

12. Baldi DL*, et al.* The type II secretion system and its ubiquitous lipoprotein substrate, SslE, are required for biofilm formation and virulence of enteropathogenic Escherichia coli. *Infect Immun* **80**, 2042-2052 (2012).
