# Supplementary material for: Molecular and cellular insight into Escherichia coli SslE and its role during biofilm maturation
Source: NPJ Biofilms Microbiomes. 2022 Feb 25;8:9. doi: 10.1038/s41522-022-00272-5 (PMC8881592; doi:10.1038/s41522-022-00272-5)
Supplement: Supplementary file 2 — Reporting Summary [file 41522_2022_272_MOESM2_ESM.pdf]

nature portfolio

Corresponding author(s): NPJBIOFILMS-01193R2

Last updated by author(s): Jan 26, 2022

Reporting Summary

Nature Portfolio wishes to improve the reproducibility of the work that we publish. This form provides structure for consistency and transparency in reporting. For further information on Nature Portfolio policies, see our Editorial Policies and the Editorial Policy Checklist.

Statistics

For all statistical analyses, confirm that the following items are present in the figure legend, table legend, main text, or Methods section.

n/a	Confirmed	
☐	☒	The exact sample size (n) for each experimental group/condition, given as a discrete number and unit of measurement
☐	☒	A statement on whether measurements were taken from distinct samples or whether the same sample was measured repeatedly
☐	☒	The statistical test(s) used AND whether they are one- or two-sided<br>*Only common tests should be described solely by name; describe more complex techniques in the Methods section.*
☒	☐	A description of all covariates tested
☒	☐	A description of any assumptions or corrections, such as tests of normality and adjustment for multiple comparisons
☐	☒	A full description of the statistical parameters including central tendency (e.g. means) or other basic estimates (e.g. regression coefficient) AND variation (e.g. standard deviation) or associated estimates of uncertainty (e.g. confidence intervals)
☐	☒	For null hypothesis testing, the test statistic (e.g. F, t, r) with confidence intervals, effect sizes, degrees of freedom and P value noted<br>*Give P values as exact values whenever suitable.*
☒	☐	For Bayesian analysis, information on the choice of priors and Markov chain Monte Carlo settings
☒	☐	For hierarchical and complex designs, identification of the appropriate level for tests and full reporting of outcomes
☒	☐	Estimates of effect sizes (e.g. Cohen's d, Pearson's r), indicating how they were calculated

Our web collection on statistics for biologists contains articles on many of the points above.

Software and code

Policy information about availability of computer code

Data collection	no software used
Data analysis	no software used

For manuscripts utilizing custom algorithms or software that are central to the research but not yet described in published literature, software must be made available to editors and reviewers. We strongly encourage code deposition in a community repository (e.g. GitHub). See the Nature Portfolio guidelines for submitting code & software for further information.

Data

Policy information about availability of data

All manuscripts must include a data availability statement. This statement should provide the following information, where applicable:

- Accession codes, unique identifiers, or web links for publicly available datasets
- A description of any restrictions on data availability
- For clinical datasets or third party data, please ensure that the statement adheres to our policy

The data that support the findings of this study are included in the article, its supplementary information files, or are available from the corresponding author upon reasonable request. The plasmids pPC1, pPC2, pPC3, pPC4, pPC5, pBD1, pCPC1 and pCPC2 have been deposited in the Addgene plasmid repository (https://www.addgene.org) with IDs 182013, 182014, 182015, 182016, 182017, 182018, 182019 and 182020, respectively. The negative stain TEM electron density map for SslE pH 7.4 has been deposited in the Electron Microscope Data Bank (https://www.ebi.ac.uk/emdb) with accession code EMD-14170. The buffer subtracted SAXS curves and DAMMIN models with the lowest NSD score for monomeric SslE at pH 4.4 and 7.4, and the SslE N1, N2 and N3-M60 domains, have been deposited in the Small Angle Scattering Biological Data Bank (https://www.sasbdb.org/) with accession ID SASDLW2, SASDLV2, SASDMU6, SASDMV6 and SASDMW6, respectively.

March 2021

Field-specific reporting

Please select the one below that is the best fit for your research. If you are not sure, read the appropriate sections before making your selection.

☒ Life sciences ☐ Behavioural & social sciences ☐ Ecological, evolutionary & environmental sciences

For a reference copy of the document with all sections, see nature.com/documents/nr-reporting-summary-flat.pdf

Life sciences study design

All studies must disclose on these points even when the disclosure is negative.

Sample size	N/A
Data exclusions	N/A
Replication	N/A
Randomization	N/A
Blinding	N/A

Reporting for specific materials, systems and methods

We require information from authors about some types of materials, experimental systems and methods used in many studies. Here, indicate whether each material, system or method listed is relevant to your study. If you are not sure if a list item applies to your research, read the appropriate section before selecting a response.

Materials & experimental systems

n/a	Involved in the study	
☐	☒	Antibodies
☒	☐	Eukaryotic cell lines
☒	☐	Palaeontology and archaeology
☒	☐	Animals and other organisms
☒	☐	Human research participants
☒	☐	Clinical data
☒	☐	Dual use research of concern

Methods

n/a	Involved in the study	
☒	☐	ChIP-seq
☒	☐	Flow cytometry
☒	☐	MRI-based neuroimaging

Antibodies

Antibodies used	Monoclonal Anti-6X His tag antibody produced in mouse, clone GT359, SAB2702219, Sigma; DsbA Monoclonal Antibody (8A10), MA5-18312, Invitrogen; Bespoke anti-SslE (rabbit) and anti-CsgA (guineapig) antibodies - polyclonal from recombinant SslE and CsgA antigens, Invitrogen
Validation	Validated by reacting with purified proteins and bacterial lysates on Western blot/immunoblotting
